# Comparison of prognostic models to predict the occurrence of colorectal cancer in asymptomatic individuals: a systematic literature review and external validation in the EPIC and UK Biobank prospective cohort studies

**DOI:** 10.1136/gutjnl-2017-315730

**Published:** 2018-04-03

**Authors:** Todd Smith, David C Muller, Karel G M Moons, Amanda J Cross, Mattias Johansson, Pietro Ferrari, Guy Fagherazzi, Petra H M Peeters, Gianluca Severi, Anika Hüsing, Rudolf Kaaks, Anne Tjonneland, Anja Olsen, Kim Overvad, Catalina Bonet, Miguel Rodriguez-Barranco, Jose Maria Huerta, Aurelio Barricarte Gurrea, Kathryn E Bradbury, Antonia Trichopoulou, Christina Bamia, Philippos Orfanos, Domenico Palli, Valeria Pala, Paolo Vineis, Bas Bueno-de-Mesquita, Bodil Ohlsson, Sophia Harlid, Bethany Van Guelpen, Guri Skeie, Elisabete Weiderpass, Mazda Jenab, Neil Murphy, Elio Riboli, Marc J Gunter, Krasimira Jekova Aleksandrova, Ioanna Tzoulaki

**Affiliations:** 1 Department of Epidemiology and Biostatistics, School of Public Health, Imperial College London, London, UK; 2 Julius Center for Health Sciences and Primary Care, Umc Utrecht, Utrecht University, Utrecht, The Netherlands; 3 International Agency for Research on Cancer (IARC), Genetic Epidemiology Group, Lyon, France; 4 Nutritional Methodology and Biostatistics Group (NMB), International Agency for Research on Cancer, Lyon, France; 5 Inserm U1018, Gustave Roussy, Universite Paris-Sud, Villejuif, France; 6 Department of Epidemiology, Julius Center for Health Sciences and Primary Care, University Medical Center Utrecht, Utrecht University, Utrecht, The Netherlands; 7 Division of Cancer Epidemiology, German Cancer Research Center (DKFZ), Heidelberg, Germany; 8 Danish Cancer Society Research Center, Copenhagen, Denmark; 9 Department of Public Health, Section for Epidemiology, Aarhus University, Aarhus, Denmark; 10 Catalan Institute of Oncology-IDIBELL, L’Hospitalet de Llobregat, Unit of Nutrition and Cancer, Cancer Epidemiology Research Program, Barcelona, Spain; 11 CIBER de Epidemiologia y Salud Publica (CIBERESP), Escuela Andaluza de Salud Publica, Madrid, Spain; 12 Murcia Regional Health Council, IMIB-Arrixaca, CIBER de Epidemiologia y Salud Publica (CIBERESP), Madrid, Spain; 13 Navarra Public Health Institute, CIBER de Epidemiologia y Salud Publica (CIBERESP), Madrid, Spain; 14 Cancer Epidemiology Unit, Nuffield Department of Population Health, University of Oxford, Oxford, UK; 15 Hellenic Health Foundation, Athens, Greece; 16 Unit of Nutritional Epidemiology and Nutrition in Public Health, Department of Hygiene, Epidemiology and Medical Statistics, School of Medicine, WHO Collaborating Center for Nutrition and Health, National and Kapodistrian University of Athens, Athens, Greece; 17 Cancer Risk Factors and Life-Style Epidemiology Unit, Cancer Research and Prevention Institute – ISPO, Florence, Italy; 18 Epidemiology and Prevention Unit, Fondazione IRCCS Istituto Nazionale dei Tumori, Milan, Italy; 19 Italian Institute for Genomic Medicine, Turin, Italy; 20 Department for Determinants of Chronic Diseases (DCD), National Institute for Public Health and the Environment (RIVM), Bilthoven, The Netherlands; 21 Department of Internal Medicine, Lund University, Skane University Hospital, Malmo, Sweden; 22 Department of Radiation Sciences, Oncology, Umea University, Umea, Sweden; 23 Department of Community Medicine, Faculty of Health Sciences, University of Tromso, The Arctic University of Norway, Tromso, Norway; 24 Department of Research, Cancer Registry of Norway, Institute of Population-Based Cancer Research, Oslo, Norway; 25 Nutritional Epidemiology Group, International Agency for Research on Cancer, Lyon, France; 26 Nutrition, Immunity and Metabolism Start-up Lab, Department of Epidemiology, German Institute of Human Nutrition, Potsdam-Rehbrucke, Germany

**Keywords:** colorectal cancer, colorectal cancer screening, cancer prevention, epidemiology, medical statistics

## Abstract

**Objective:**

To systematically identify and validate published colorectal cancer risk prediction models that do not require invasive testing in two large population-based prospective cohorts.

**Design:**

Models were identified through an update of a published systematic review and validated in the European Prospective Investigation into Cancer and Nutrition (EPIC) and the UK Biobank. The performance of the models to predict the occurrence of colorectal cancer within 5 or 10 years after study enrolment was assessed by discrimination (C-statistic) and calibration (plots of observed vs predicted probability).

**Results:**

The systematic review and its update identified 16 models from 8 publications (8 colorectal, 5 colon and 3 rectal). The number of participants included in each model validation ranged from 41 587 to 396 515, and the number of cases ranged from 115 to 1781. Eligible and ineligible participants across the models were largely comparable. Calibration of the models, where assessable, was very good and further improved by recalibration. The C-statistics of the models were largely similar between validation cohorts with the highest values achieved being 0.70 (95% CI 0.68 to 0.72) in the UK Biobank and 0.71 (95% CI 0.67 to 0.74) in EPIC.

**Conclusion:**

Several of these non-invasive models exhibited good calibration and discrimination within both external validation populations and are therefore potentially suitable candidates for the facilitation of risk stratification in population-based colorectal screening programmes. Future work should both evaluate this potential, through modelling and impact studies, and ascertain if further enhancement in their performance can be obtained.

**Significance of this study**

**What is already known on this subject?**

- Whereas risk prediction models are commonly used in clinical practice, none are widely used for colorectal cancer. Accurately identifying people at increased risk of developing colorectal cancer would provide substantial public health benefits.
- There are a range of existing models, based on non-invasive variables, to predict the future occurrence of colorectal cancer. To our knowledge, they have not yet all been externally validated and evaluated in comparable populations.
- External validation is essential for the selection and optimisation of the best models, aiding their potential for future implementation in clinical practice.

**What are the new findings?**

- This comprehensive external validation and comparison of non-invasive colorectal cancer risk prediction models in two large population-based cohorts provides a basis for their future selection and development for clinical practice.
- The models were well calibrated in EPIC and the UK Biobank, with further improvements achieved after recalibration. The discrimination of the models was similar between the two cohorts, with C-statistics of up to 0.71.

**Significance of this study**

**How might it impact on clinical practice in the foreseeable future?**

- The good calibration and discrimination observed in the better performing models provides evidence that further work (including modelling and impact studies) should be undertaken to assess their potential clinical utility.

## Introduction

Colorectal cancer accounts for 10% of all cancers in men and 9% in women worldwide, representing the third and second most common cancer types, respectively, with an estimated combined annual incidence of over 1.3 million cases and 694 000 deaths.^1^ Population-based screening strategies, which many countries have implemented or are in the process of implementing,^2^ have the potential to reduce this substantial burden.^3^

Risk prediction models that estimate the probability of developing colorectal cancer in an asymptomatic, community-dwelling, population could substantially improve the efficiency and implementation of these population-based screening strategies by facilitating risk-stratified approaches. Additionally, as has been demonstrated in cardiovascular disease,^4^ these models can also be used to target prevention strategies and/or as a tool to aid risk communication.

Several models exist for predicting the risk of developing colorectal, colon or rectal cancer in asymptomatic populations.^5^ However, little is known about the comparative ability of these models to identify future cases when validated head to head in the same population. This information is essential to guide which models should be considered for further optimisation or for assessment of their clinical utility and implementation.

Risk prediction models that incorporate information that is routinely available or easily ascertained without ‘invasive’ tests are particularly attractive as they are easier to implement in a general population or primary care screening setting. Therefore, we performed, to our knowledge, the first systematic comparison and external validation of published ‘non-invasive’ colorectal cancer risk prediction models in two large independent prospective cohort studies, which collectively represent just under 1 million individuals.

## Methods

### Systematic literature search and identification of published models

We extended a previously published systematic review of colorectal cancer models from March 2014 to July 2016, using the same search algorithm.^5^ Studies extracted from the previous systematic review and newly identified studies from our updated literature search were included if: the study presented at least one formal prediction model; it was developed to provide individualised predictions; the endpoint was incident colorectal cancer or a subsite within it; the population was a general population or community-dwelling setting, not a specific symptomatic, clinical or high-risk patient population group; predictors in the model were measurements that could be taken non-invasively (through questionnaire, physical measurements and so on); and predictors only had to be measured at a single point in time rather than repeated longitudinal measurements. In addition, any updates or validations of identified models that continued to meet the selection criteria were retained. Only full articles (ie, not conference abstracts) and articles published in English were considered eligible.

Of those studies identified in the initial systematic review,^5^ TS reviewed their summaries and referred to individual abstracts and full texts where required. Those with the potential to be included were reviewed by a second author (DCM/IT). In the extension to the systematic review, the search results were collated in the reference management software Endnote X7. After the removal of duplicates, the resultant publications’ titles and abstracts were screened by TS, and those deemed to have the potential to contain eligible models were retained. These were then independently reviewed by two authors (TS and DCM/IT) to identify those papers containing eligible models, using the full text where available and required. Any discrepancies between reviewers were resolved by consensus. In addition, the reference list of any eligible study identified through the aforementioned processes was reviewed to check for unidentified models. As the intention of this systematic review was to identify models for subsequent external validation, a risk of bias assessment was not undertaken.

### Data extraction

From each eligible risk prediction model we extracted the necessary information to perform an external validation, following the applicable guidance from the checklist for critical appraisal and data extraction for systematic reviews of prediction modelling studies (CHARMS)^6^: first author’s name, year of publication, country, number of cases and population size, outcomes examined, age range of participants, duration of follow-up, statistical model, number of predictors, definition of each predictor (including thresholds for categorical predictors), reported performance of the model, reported performance in internal or external validation (if done) and the parameter estimates, including predictors relative risks or coefficients. If a subsequently published validation of a model incorporated either an updated predictor definition or coefficient value then these were substituted for the original. Where models estimated absolute risks, then additional data to assess the calibration of the model was also recorded including age-specific cancer hazard rates,^7 8^ age-specific mortality rates,^7 8^ attributable risks,^7^ survivor functions,^9 10^ mean values for each risk factor in the cohort^9^ and the risk score estimated at the means of all predictors.^10^

### Validation cohorts

We used two large, multicentre, population-based cohorts to validate the eligible colorectal cancer risk prediction models. The European Prospective Investigation into Cancer and Nutrition (EPIC) is a multicentre prospective cohort study comprising 521 324 participants aged 17 to 98 years at baseline (though predominantly 35–70 years) who were recruited between 1992 and 2000 across 23 centres in 10 European countries.^11^ Participants were enrolled from a variety of sources and included blood donors, screening participants, health conscious individuals and the general population (more detailed information regarding the characteristics and eligibility criteria of the individual centres has been published elsewhere).^11^ Baseline data on each participant’s diet and lifestyle were generally collected through self-completed questionnaires with anthropometric measurements being recorded subsequently at a recruitment centre; however, there was variability between centres.^11^ After the exclusion of individuals with prevalent cancer at recruitment (except for non-melanoma skin cancers), there were 491 992 available participants in EPIC with censoring due to the end of follow-up ranging from 28 June 2008 (France) to 31 December 2013 (Sweden). Colorectal cancer diagnoses were identified by a number of methods including cancer registries, health insurance records, pathology registries and active follow-up.^12^

The UK Biobank is a multicentre prospective cohort of over 500 000 participants aged 40–69 years at baseline who were identified through National Health Services registers and recruited between 2006 and 2010, across 22 assessment centres in Great Britain.^13 14^ Baseline data recorded at the assessment centres included information about diet and lifestyle along with anthropometric measures, obtained through a combination of a self-completed questionnaire, computer-assisted interview and physical measurement.^14^ From an initial total of 502 639 participants, 475 629 were available for external validation after the exclusion of participants with cancers prevalent at recruitment (except non-melanoma skin cancers). We set the end of follow-up for cancer incidence as the 1st January 2015. Colorectal cancer diagnoses within the cohort’s participants was ascertained from national cancer registries.^15^

### Statistical analysis

#### Model predictors, outcomes and time horizon

We first attempted to match the predictors of the original models with the variables available in EPIC and the UK Biobank cohorts. When a direct match could not be achieved but a suitable surrogate(s) existed, we defined it as closely as possible to the model’s original predictor definition. Online supplementary table 1 provides a description of the variables used in each of the prediction models and details of the redefinition when it was required. In cases where variables or suitable surrogates were unavailable for a substantial majority or the entirety of the cohort, we assumed a single value chosen to be typical of the population and applied it to all participants. For instance, in the EPIC cohort, all individuals were set to be non-users of non-steroidal anti-inflammatory drugs when a model incorporated this variable.

10.1136/gutjnl-2017-315730.supp1 Supplementary file 1

In the presence of other missing predictor data, where it was less restrictive on the resultant size of the validation population, we conducted complete case analysis. As several of the models evaluated were time-to-event models, imputation was not undertaken as it tends to produce biased estimates.^16^ The number of participants contributing to the evaluation of each model thus differed depending on the extent of missing data in its constituent predictors.

Participants aged between 40 and 70 years at recruitment were included in the validation of all models, unless the model was developed in an older population, in which case we matched the minimum age of the original population.

The eligible and ineligible populations for each model were contrasted, by the cumulative incidence of the cancer(s) the model predicted, the age of the participants and the presence of five modifiable risk factors: body mass index (BMI),^17^ smoking,^18^ alcohol intake,^17 18^ physical activity^17^ and processed meat consumption.^17^

The outcome predicted for all models tested was the incidence of a first primary colorectal cancer or cancer at an anatomical subsite within the colorectum (International Statistical Classification of Diseases and Related Health Problems 10th Revision codes: C18 (except C18.1, Appendix), C19 and C20), within the time frame of the model prior to censoring by another cancer diagnosis, death or end of follow-up.

Based on the current length of follow-up in the two cohorts, the prediction horizon for the models was restricted to a maximum of 10 years in EPIC and 5 years in the UK Biobank.

#### Model performance

We assessed the discrimination and calibration of all eligible models in the two cohorts separately. The discrimination of each model was assessed using the concordance (C)-statistic and its 95% CI. This was initially calculated as Somers’ D^19^ and then transformed into Harrell’s C-statistic^20^ ((Somers’ D+1)/2).^21^ The values achieved by each model in EPIC and the UK Biobank were then compared.^22^ For the Colditz model neither the age and sex specific incidences nor the population prevalences were provided in full in the original paper. To obtain absolute risks we calculated these in the validation cohorts, as a result the estimates of discrimination correspond to a recalibrated model.^23^ Calibration of the original models and their subsequent recalibration was assessed graphically by plotting the mean observed probability against the mean predicted probability within tenths of the predicted probabilities. Where authors produced sex-specific models, these were first combined by amalgamating their individual absolute risk values. In order to derive the absolute risk estimate required for the assessment of calibration, we applied the full prediction rule of the original models, as they were published, to our two study cohorts. When this was not available, in models that were constructed to provide an absolute risk, we contacted the authors to obtain this information. If this was unsuccessful, we only provide information on the recalibration of these models. Recalibration was undertaken for the logistic regression-based models by refitting the model intercept in the validation cohorts along with their published predicted log-odds as an offset. While in the survival model-based risk models, we estimated the baseline survival function in the validation cohorts and combined this with the predicted hazard ratios from the published model to obtain recalibrated predicted probabilities.

Resultantly, this meant we were able to provide calibration plots for five of the eight publications in EPIC and three of the eight publications in the UK Biobank. Three calibrations were not possible to assess in either cohort, either because the model was not constructed to provide an absolute risk^24^ or because the required data could not be obtained.^23 25^ A further two^9 26^ were not possible to assess in the UK Biobank as the baseline survival function provided was for a time horizon of 10 years, while in this cohort, due to the length of follow-up available, they were assessed over 5 years. In these cases, we present recalibrated estimates only.

In the UK Biobank, common protocols and assessment procedures were used for all study participants; therefore, it was treated as a single population in all analyses. However, as EPIC encompasses diverse geographical locations across multiple Europe countries, we calculated model discriminations in each country separately (n varied from 3 to 9) and then used meta-analysis to summarise the results after logit transformation.^27^ This approach also provided a measure of heterogeneity between countries (I^2^).

Stata V.13 software and user-written packages^19 28–32^ were used to construct and validate each model either in full or where code was available for alternative software in part (one publication^7^ had an associated macro^33^ for which we used SAS software (V.9.4)).

#### Patient involvement

Patients were not involved in the study design.

## Results

### Identification of models for inclusion in the systematic review

Online supplementary figure 1 shows the flow of the study selection process. From the systematic review,^5^ we selected seven publications that contained at least one eligible model and two publications that documented external validation of these models. Our updated literature search yielded 7914 publications, of which 116 publications were selected for full-text review, and one publication was deemed eligible. Collectively, these two search strategies resulted in the identification of eight publications, containing 16 eligible models, originating from a diverse range of derivation populations (online supplementary table 2).

10.1136/gutjnl-2017-315730.supp2 Supplementary file 2

### Characteristics of the included models

Of the 16 eligible models (table 1), eight examined the risk of colorectal cancer, five the risk of colon cancer and three the risk of rectal cancer, specifically. Twelve were sex-specific risk models (of which six were paired male and female models, for the same anatomical site, from the same publication) and four incorporated both sexes. Age was included in all models, either as a covariate or through the use of age-specific rates, with other common covariates being BMI (13 models), alcohol consumption (13 models) and smoking (9 models). The number of predictors ranged from 2^8^ to 14.^23^ All female-specific models incorporated some indication of oestrogen exposure,^7 23 26^ with the exception of the model published by Shin *et al*.^25^ In their original publications, the model’s performance, or illustrative examples, had been reported over time horizons ranging from 5 to 20 years (table 2). All models were validated over 5 years in the UK Biobank, given the limited follow-up time in the cohort. In EPIC they were validated over 10 years unless they had been developed as 5-year models,^10 25^ in which case this time horizon was used.

**Table 1:** Predictor variables contained within the 16 identified eligible colorectal cancer risk prediction models

Author	Sex	Outcome	Predictor variables																			
			Demographic characteristics				Anthropometry		Family history of Cancer	Medical history				Medication use			Lifestyle factors			Diet		
			Sex	Age	Eth	Edu	Height	BMI		Diabetes	Screen/endo/ polyp	IBD	Menopausal status	HRT	OC	NSAID	Physical activity	Smoking	Alcohol	Red meat/ meat	Veg	Vit
Colditz^23^	M	C		●			●	●	●		●	●				●	●		●	●	●	●
	F	C		●			●	●	●		●	●		●	●	●	●		●	●	●	●
Driver^24^	M	CRC		●				●										●	●			
Freedman^7^	M	CRC		●				●	●		●					●	●	●			●	
	F	CRC		●				●	●		●		○ α	○ α		●	●				●	
Ma^9^	M	CRC		●				●									●	●	●			
	M	C		●				●									●	●	●			
	M	R		●													●		●			
Shin^25^	M	R C		●				●	●										●	●		
	F	R		●			●												●	●		
Steffen^10^	Both	CRC	●	●				●		●	●							●	●			
	Both	C	●	●				●		●	●							●	●			
	Both	R	●	●				●		●	●							●	●			
Taylor^8^	Both	CRC		●					●													
Wells^26^	M	CRC		●	●	●		●	●	●						●	●	●	●	●		●
	F	CRC		●	●	●		●	●	●				○ µ	○ µ	●		●	●			●
Variable availability in EPIC			■	■		□	■	■	□	■	□		na	□	□	□	■	■	■	□	□	□
Variable availability in UK Biobank			■	■	■	□	■	■	□	■	■	■	na	□	□	□	□	■	■	□	□	■

●, variable included in the original model; ○, two variables amalgamated to create a new variable that was included in the model. The names of these variables were: α, oestrogen status; µ, oestrogen. Family history of cancer varied by both cancer site and degrees of relatedness between models. ■, available in a construction directly suitable for at least one model; □, variable required to be derived from other variable(s) in the dataset for all models that used it; na, not applicable as the variable is amalgamated in the model in which it is used. See online supplementary table 1 for further details.

BMI, body mass index; C, colon cancer; CRC, colorectal cancer; Edu, years of education; EPIC, European Prospective Investigation into Cancer and Nutrition; Eth, ethnicity; F, female; HRT, hormone replacement therapy; IBD, inflammatory bowel disease; M, male; NSAID, non-steroidal anti-inflammatory drug; OC, oral contraceptive; R, rectal cancer; RC, right colon cancer; screen/endo/polyp, history of colorectal cancer screening or lower gastrointestinal endoscopy with or without identification of polyps; Veg, vegetables; Vit, multivitamin.

**Table 2:** Colorectal cancer risk model discrimination in the published literature, EPIC and the UK Biobank

Author	Sex	Site	Time horizon (years)	Derivation C-statistic (95% CI)	Published validation C-statistic* (95% CI)	UK Biobank C-statistic (95% CI)	UK Biobank Sex-combined C-statistic (95% CI)	EPIC C-statistic (95% CI)	EPIC Sex-combined C-statistic (95% CI)	I^2^statistic across EPIC countries (%)
Colditz^23^	Male	Colon	10		0.71 (0.68 to 0.74)^42^	0.68 (0.66 to 0.70)	0.67 (0.65 to 0.68)	0.67 (0.64 to 0.70)	0.66 (0.64 to 0.69)	62.8
	Female	Colon			0.67 (0.64 to 0.70)^42^	0.63 (0.60 to 0.65)		0.65 (0.62 to 0.69)		62.3
Driver^24^	Male	Colorectal	20	0.695		0.68 (0.67 to 0.69)		0.67 (0.64 to 0.70)		77.6
Freedman^7^	Male	Colorectal	10 and 20		0.61 (0.60 to 0.62)^43^	0.60 (0.58 to 0.62)	0.61 (0.59 to 0.62)	0.61 (0.59 to 0.63)	0.61 (0.59 to 0.62)	0.0
	Female	Colorectal			0.61 (0.59 to 0.62)^43^	0.58 (0.56 to 0.61)		0.58 (0.56 to 0.60)		0.0
Ma^9^	Male	Colorectal	10	0.70 (0.68 to 0.72)	0.64 (0.61 to 0.67)	0.69 (0.68 to 0.71)		0.68 (0.65 to 0.70)		65.9
	Male	Colon		0.71 (0.68 to 0.74)	0.66 (0.62 to 0.70)	0.70 (0.68 to 0.72)		0.69 (0.66 to 0.72)		51.9
	Male	Rectal		0.68 (0.64 to 0.71)	0.62 (0.57 to 0.66)	0.68 (0.65 to 0.70)		0.66 (0.63 to 0.68)		1.3
Shin^25^	Male	Right Colon	5	0.74 (0.72 to 0.76)	0.76 (0.73 to 0.79)	0.68 (0.65 to 0.71)		0.71 (0.67 to 0.74)		0.0
	Female	Rectal		0.70 (0.68 to 0.71)	0.72 (0.70 to 0.74)	0.63 (0.59 to 0.67)		0.62 (0.58 to 0.67)		37.7
Steffen^10^	Both	Colorectal	5	0.73 (0.72 to 0.74)	0.70 (0.66 to 0.73)	0.68 (0.67 to 0.69)		0.68 (0.65 to 0.71)		68.1
	Both	Colon		0.75 (0.73 to 0.76)	0.72 (0.68 to 0.76)	0.69 (0.67 to 0.70)		0.69 (0.66 to 0.72)		45.4
	Both	Rectal		0.73 (0.71 to 0.76)	0.64 (0.58 to 0.70)	0.66 (0.64 to 0.68)		0.64 (0.61 to 0.68)		33.0
Taylor^8^	Both	Colorectal	20	0.64		0.67 (0.66 to 0.68)		0.67 (0.65 to 0.69)		0.0
Wells^26^	Male	Colorectal	10	0.681 (0.669 to 0.694)		0.69 (0.67 to 0.71)	0.67 (0.65 to 0.68)	0.70 (0.67 to 0.73)	0.69 (0.67 to 0.71)	20.9
	Female	Colorectal		0.679 (0.665 to 0.692)		0.62 (0.60 to 0.64)		0.67 (0.65 to 0.70)		0.0

*Validation C-statistics were obtained from the original derivation publications or, where indicated, from validation studies identified in the systematic review.

EPIC, European Prospective Investigation into Cancer and Nutrition.

### Characteristics of the validation populations

The number of individuals included in each validation varied across both the models and the cohorts due to the availability of their predictors (online supplementary table 1). Within EPIC, the percentage of eligible participants included ranged from 17.1% (n=25 273)^8^ to 84.0% (n=124 293)^25^ for male participants and 20.1% (n=69 154)^26^ to 60.4% (n=207 887)^25^ for female participants, while for the UK Biobank, these values were 42.7% (n=93 608)^26^ to 98.7% (n=216 440)^24^ and 45.8% (n=117 367)^26^ to 85.7% (n=219 484),^8^ respectively (online supplementary tables 3–6). As some models included both sexes, this meant that actual model population sizes ranged from 41 587^26^ to 396 515^8^ and the number of cases per model ranged from 115^25^ to 1781.^10^ There was variability in how closely matched the eligible and ineligible populations were across cancer incidence rate, age and modifiable risk factors; however, overall, they were largely comparable.

### External validation

**Figure 1:**
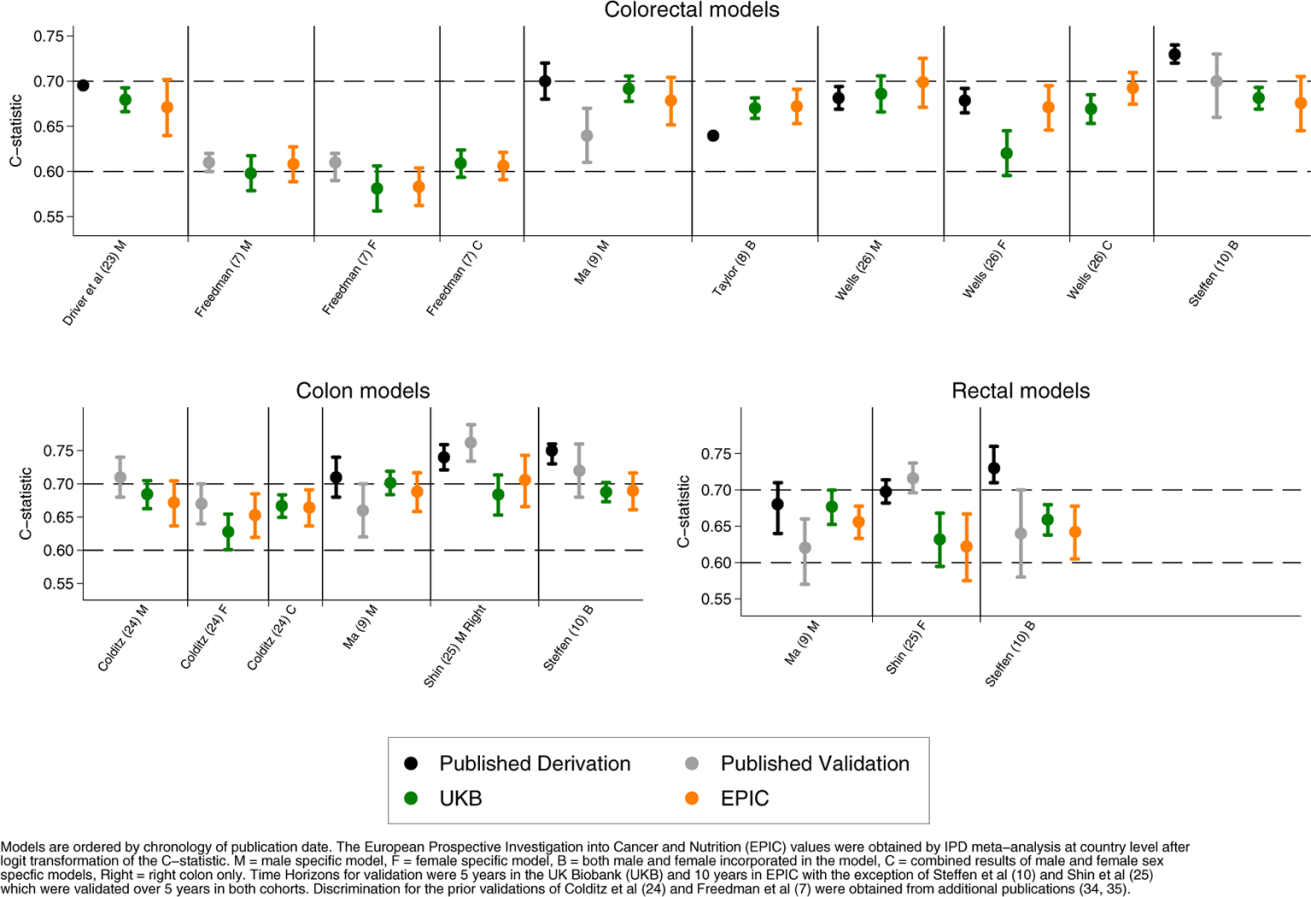
Discrimination of colorectal cancer risk prediction models by anatomical location.

Figure 1 shows the C-statistics of the models in the UK Biobank and EPIC as well as their previously reported derivation and validation values where they were available. On the whole the models showed similar levels of estimated discrimination between EPIC and the UK Biobank with the exception of the female Wells *et al* model,^26^ which had higher discrimination in EPIC (EPIC C-statistic 0.67 (95% CI 0.65 to 0.70), UK Biobank C-statistic 0.62 (95% CI 0.60 to 0.64)) (table 2). In the UK Biobank, the C-statistic varied between 0.58 (95% CI 0.56 to 0.61) (Freedman *et al*’s female colorectal model^7^) to 0.70 (95% CI 0.68 to 0.72) (Ma *et al*’s colon model^9^). In EPIC, the C-statistic varied between 0.58 (95% CI 0.56 to 0.60^7^ (Freedman *et al*’s female colorectal model^7^) to 0.71 (95% CI 0.67 to 0.74) (Shin *et al*’s male right colon model^25^).

The highest C-statistic achieved in the validation by a colorectal model was 0.70 (95% CI 0.67 to 0.73) (Wells *et al* male model^26^ in the EPIC cohort); this was also the highest value achieved by a colon cancer model that encompassed the entire colon (0.70, 95% CI 0.68 to 0.72, Ma *et al*^9^ in the UK Biobank). For rectal models, the highest C-statistic was 0.68 (95% CI 0.65 to 0.70) (Ma *et al*^9^ for men in the UK Biobank). These models were all male specific.

Of the female-specific models, the Wells *et al* model^26^ achieved the highest colorectal cancer discrimination with a C-statistic of 0.67 (95% CI 0.65 to 0.70 in EPIC). There was only one female-specific colon cancer and one female-specific rectal cancer model to be validated; the highest values obtained between the two cohorts was 0.65 (95% CI 0.62 to 0.69) (Colditz *et al*^23^ in EPIC) and 0.63 (95% CI 0.59 to 0.67) (Shin *et al*^25^ in the UK Biobank), respectively.

Finally, when assessing models that incorporated both sexes or where the results of sex-specific models were merged, the model(s) with the highest colorectal discrimination were by Wells *et al*^26^ with a C-statistic of 0.69 (95% CI 0.67 to 0.71) in the EPIC cohort.

The I^2^ obtained for each model, from the meta-analysis of the available constituent countries in the EPIC cohort, ranged from 0.0%^7 8 25 26^ to 77.6%.^24^ Six of the 16 models, representing four publications,^9 10 23 24^ had I^2^ that exceeded 50%.

**Figure 2:**
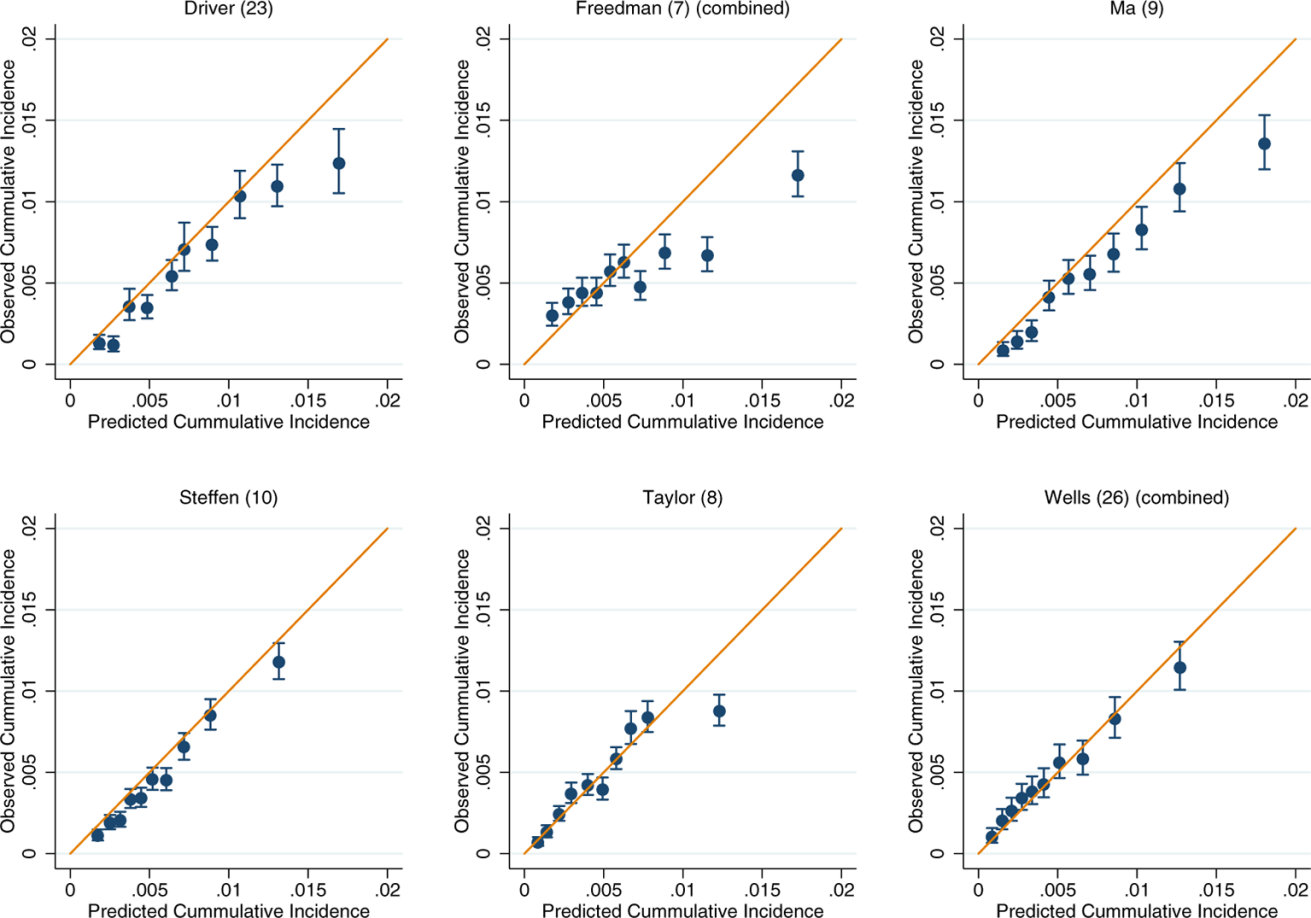
Recalibration plots of colorectal cancer risk models within the UK Biobank. Time horizon was 5 years for all models.

**Figure 3:**
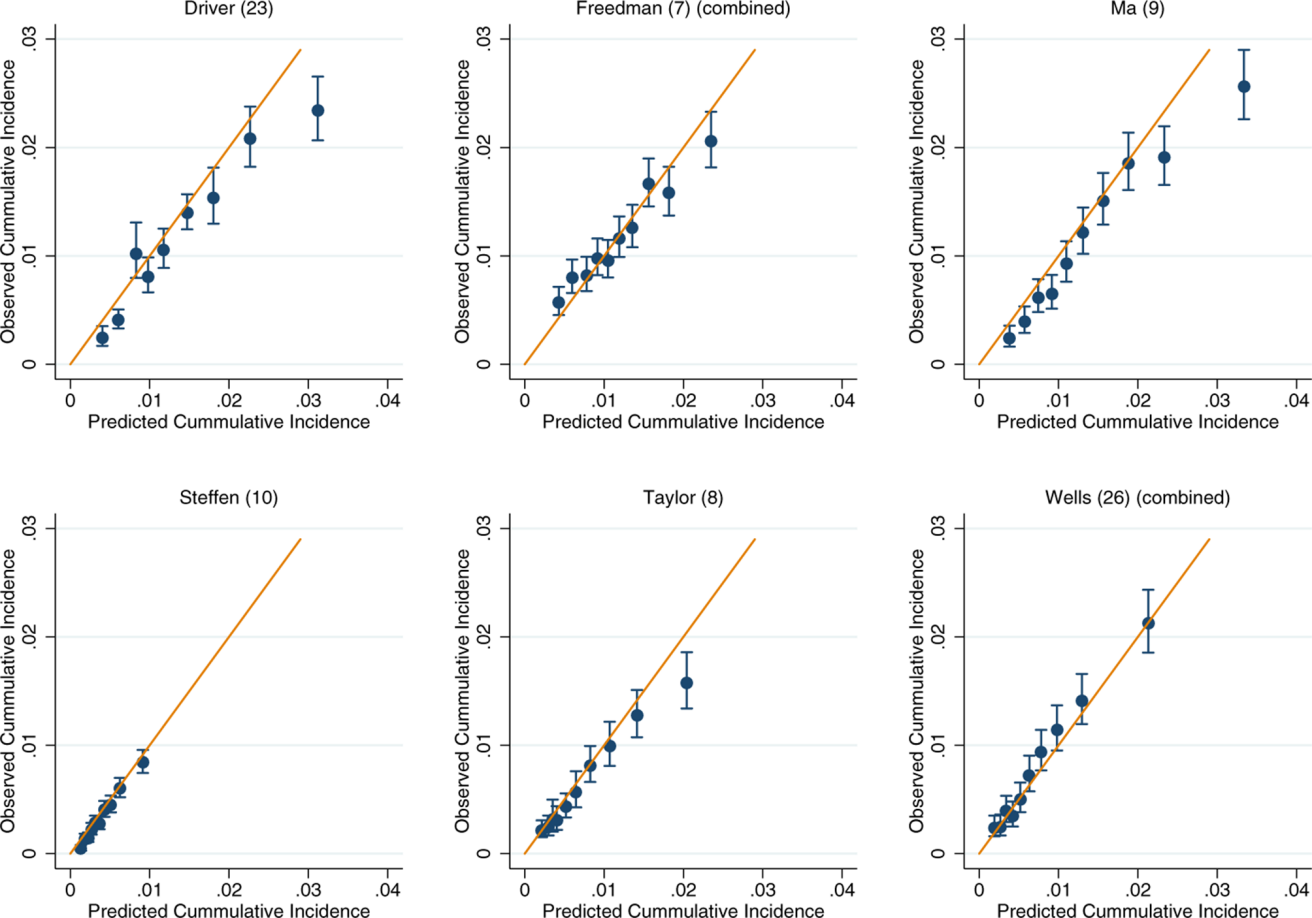
Recalibration plots of colorectal cancer risk models within the European Prospective Investigation into Cancer and Nutrition. Time horizon was 10 years for all models except Steffen *et al* which was 5 years.^10^

**Figure 4:**
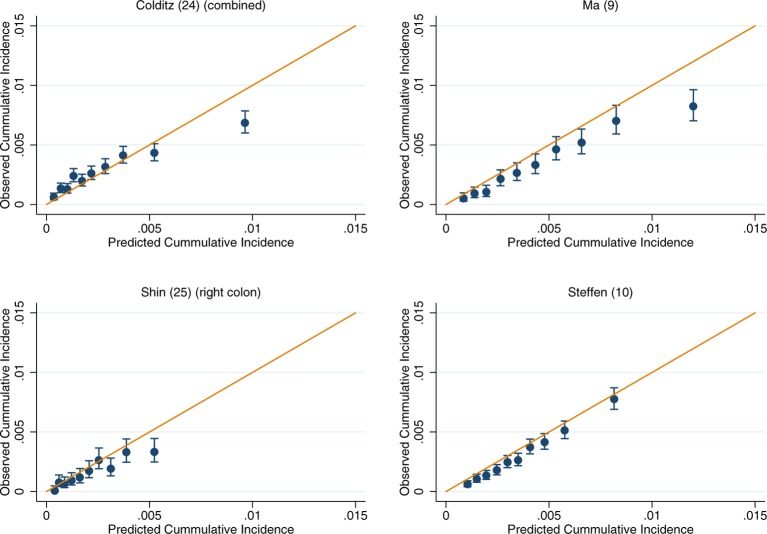
Recalibration plots of colon cancer risk models within the UK Biobank. Time horizon was 5 years for all models.

**Figure 5:**
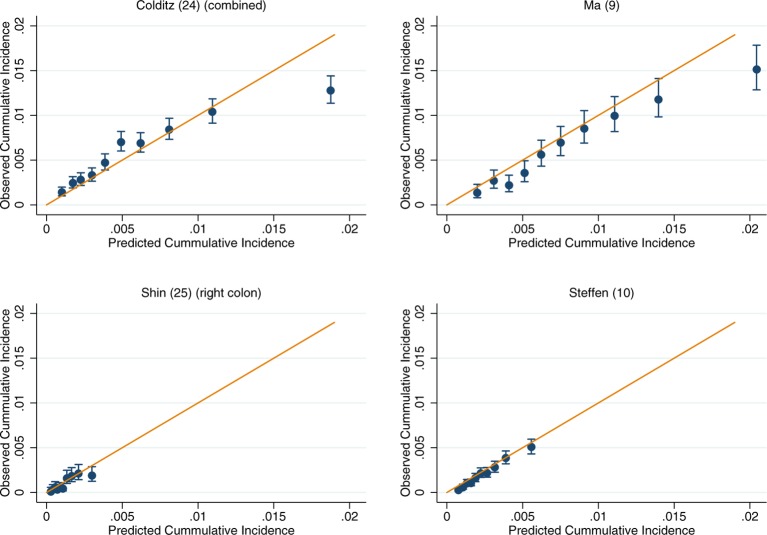
Recalibration plots of colon cancer risk models within the European Prospective Investigation into Cancer and Nutrition. Time horizon was 10 years for all models except Shin *et al* and Steffen *et al* which was 5 years.^10 25^

**Figure 6:**
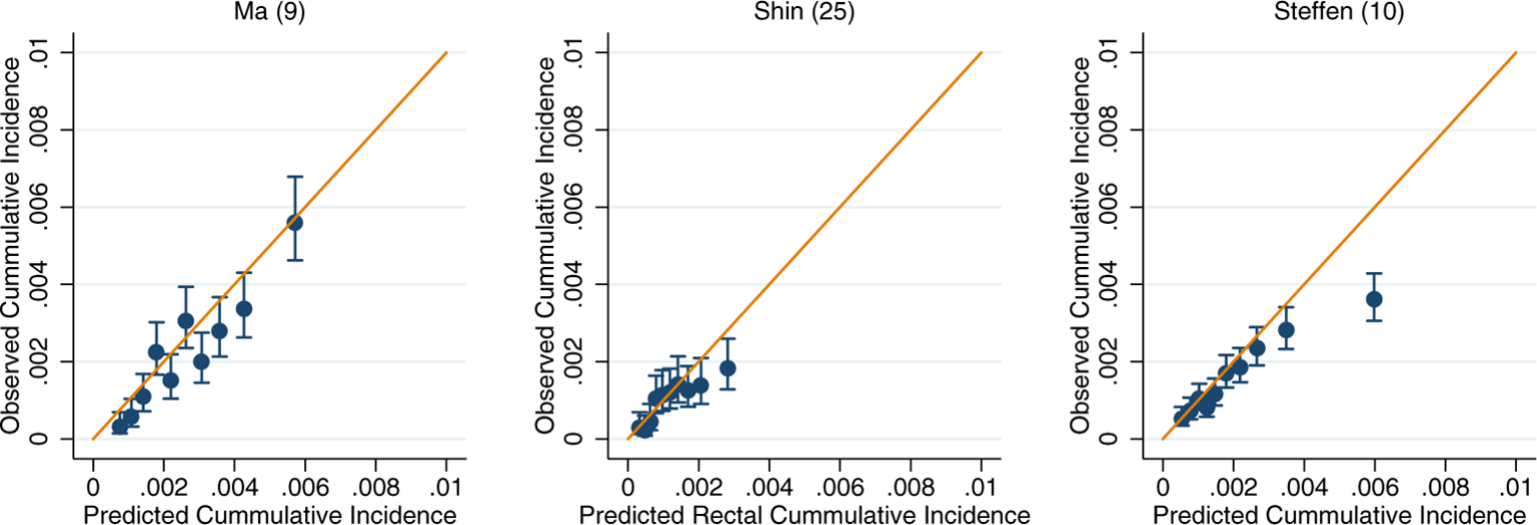
Recalibration plots of rectal cancer risk models within the UK Biobank. Time horizon was 5 years for all models.^10 25^

**Figure 7:**
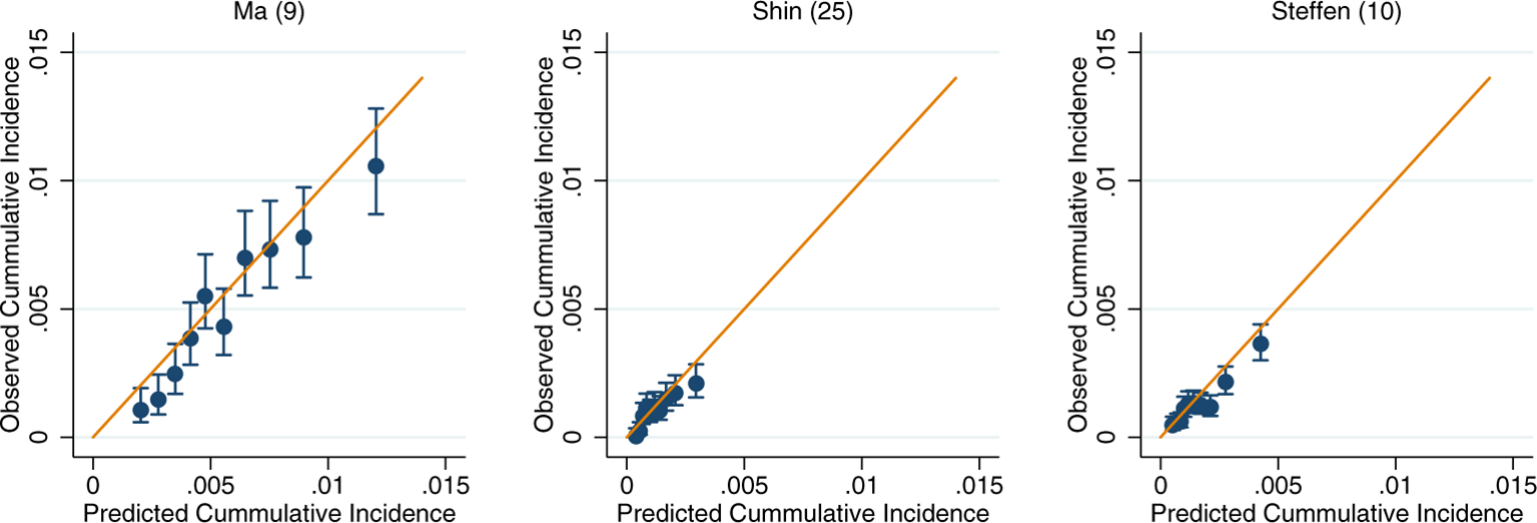
Recalibration plots of rectal cancer risk models within the European Prospective Investigation into Cancer and Nutrition. Time horizon was 10 years for Ma et al and 5 years for Shin *et al* and Steffen *et al*.^9 10 25^

For those models for which calibration could be assessed, there was good performance across both the UK Biobank and EPIC (online supplementary figures 2-7). Within the colorectal models, the slight exceptions were Freedman *et al*’s model^7^ in the UK Biobank and Ma *et al*’s model^9^ in EPIC, which show some overestimation at the upper deciles of observed cancer incidence (online supplementary figures 2 and 3). After recalibration of the models to the populations in which they were being validated in, the performance of suboptimally calibrated models was improved. Across all models, close agreement between the predicted and observed risk was observed, with only on occasion the uppermost 10th of predicted risk overestimating the observed risk (figure 2–7). The only exception was Freedman *et al*’s model,^7^ which showed notable over prediction for those in the highest 20% of predicted risk in the UK Biobank (figure 2).

## Discussion

We conducted an external validation of 16 colorectal, colon and rectal cancer risk prediction models with easy-to-measure predictors in two large European cohort studies and compared their predictive performances. Overall, the models exhibited good calibration, better for example than what was achieved across an external validation of type 2 diabetes models^34^ and performed well in discriminating between those individuals who were subsequently diagnosed with colorectal cancer and those that were not, with over 70% of the C-statistic estimates ranging between 0.65 and 0.71. The majority of this discriminative ability, among models that incorporated the whole colorectum, was achievable from using just age and family history as in the Taylor *et al* model^8^ (C-statistic of 0.67). Colorectal models that improved on this and incorporated both sexes (or where sex-specific models could be merged) required substantially more predictors, 7 for a C-statistic of 0.68 in Steffen *et al*^10^ (sex, age, BMI, diabetes, colorectal cancer screening, smoking and alcohol consumption) and 13 for C-statistics of 0.67 in the UK Biobank and 0.69 in EPIC in Wells *et al*^26^ (age, ethnicity, education, BMI, family history, diabetes, oestrogen exposure, non-steroidal anti-inflammatory use, physical activity, smoking, alcohol, red meat intake and multivitamin use).

### Clinical application

Risk prediction models with good calibration could improve the efficiency and implementation of screening programmes by targeting screening and screening intensity to those at greatest risk. Our findings provide support for the further evaluation of several of the models we identified, in this context, and would suggest in the first instance that this should incorporate both modelling and impact studies. Given the close performance between the Steffen *et al*^10^ and Wells *et al*^26^ models, there is little to choose between the two and both should be considered given the availability of their predictors. Furthermore, the similar performance of the Taylor *et al*^8^ model, along with its requirement for only two predictors (age and family history of colorectal cancer), provides a strong case for its inclusion despite its continued overestimation of risk in the uppermost decile of predicted risk after recalibration. Additionally, given the use of flexible sigmoidoscopy in screening settings,^35^ there is a potential for the use of a right colon model like Shin *et al*’s^25^ to identify those individuals who may be better served by a colonoscopy rather than a sigmoidoscopy.

An additional challenge faced by colorectal screening programmes is the population uptake of the screening test. In England in 2015/2016, the percentage uptake for bowel cancer screening within 6 months of invitation for those aged 60–74 years was only 56.4%.^36^ This low participation rate is a substantial public health challenge and needs to be addressed to maximise the benefits that can be achieved by population-based screening. Well-calibrated risk prediction models could play a role in this, particularly among the higher risk population, by facilitating an awareness of personalised risk estimates. The potential of this approach has been highlighted by an English-based study examining the association between an individual’s perceived risk of colorectal cancer with their interest in screening, finding that those who believed they had a higher than average chance of developing colorectal cancer had a greater interest in screening (98%) than those who believed they had an average (84%) or lower than average (74%) chance.^37^ Although it was not assessed if this translated into actual screening attendance, a recent meta-analysis has reported a z-score of 0.13 (95% CI 0.10 to 0.16) for the association between colorectal cancer risk perception and reported screening behaviour,^38^ and given the substantial numbers currently not participating in colorectal screening, a small improvement may yet provide a substantial benefit to the population. Finally, if chemopreventative agents are adopted for use in the general population, colorectal cancer prediction models could play a part in facilitating the identification of those for whom the benefits of treatment are more likely to outweigh possible harms.

### Strengths and limitations of the study

This is, to our knowledge, the first study to collate and externally validate non-invasive colorectal cancer risk prediction models in two large prospective cohorts. The systematic nature of the model identification, the large prospective sample sizes in which the models were validated and the opportunity to contrast the performance between cohorts are all substantial strengths. Although the time horizon for most models varied between the cohorts, this does not appear to be a limiting factor, as highlighted by their concordance in performance, but it cannot be excluded. The main limitation was the incomplete nature of several of the variables in the cohorts that necessitated in some cases in having to drop individuals from the analysis while in others, where this represented a substantial proportion of the cohort (as was seen in EPIC), a need to generalise a representative value across the entire cohort. Because of this, the models were not validated in identical samples. This raises several methodological challenges. In the first instance, the omission of individuals without a complete set of variables has the potential to limit the comparability of the models, particularly in EPIC where data availability can vary geographically. However, on contrasting the characteristics between eligible and ineligible participants across models (online supplementary table 3), the main characteristics were observed to vary little between the subgroups, and the validation samples for each model were largely comparable. The second challenge, where a single value was applied for all participants in EPIC for a given variable, may lead to a reduction in discrimination. In most instances, the effect of this strategy in the present study will be small. For example, Colditz *et al*^23^ defined aspirin use as a binary variable with a threshold at daily use for 15 years or more. As a result, the number of participants who would have met this criterion (if known) is likely to be small and so at the population level this is unlikely to have provided much additional discrimination. Furthermore, both Colditz *et al*^23^ and Steffen *et al*^10^ incorporated a history of colorectal cancer screening into their models. However, as EPIC completed its recruitment in 2000, prior to the advent of colorectal cancer screening in most of its constituent countries,^2^ setting all participants to being unscreened is likely to be an accurate representation. Even where the variable definition or cohort time frame does not provide support for the application of a single value for all participants, for example, family history of colorectal cancer, the similarity of the discriminative value to that achieved in the UK Biobank (figure 1) is suggestive that the assumptions are not likely to have strongly affected our estimates of discrimination.

The issue of missing data in the application of risk prediction models is not straightforward to resolve.^39^ While it is reasonable to apply multiple imputation techniques when developing a model, it is not clear what one should do when attempting to apply a model to an individual patient who is missing data on one or more predictors and for which multiple imputation may be impractical or impossible. One promising approach is based on fitting, in the model development phase, Pattern Mixture Kernel Submodels for each pattern of missing data.^40^ Further research on this and other methods for dealing with missing predictors at the implementation stage is urgently required if risk prediction models are to be used optimally as part of routine care and medical decision making.

## Conclusion

Our systematic approach has externally validated a range of non-invasive colorectal cancer risk prediction models across two large prospective cohorts and provided a valuable insight into their performance. We identified several models, including Steffen *et al*,^10^ Wells *et al*^26^ and Shin *et al*,^25^ with good discrimination and quantification of the actual risk of colorectal cancer, providing promise for their clinical utility in a prevention setting. This demonstrates that we are now at point where we should, through impact studies, assess the clinical utility of these better performing models as well as examine the value of incorporating additional predictors into them, as has already been called for in other areas of risk prediction.^41^ For example, incorporating risk prediction models into screening programmes could be useful for more refined, risk-based guidelines for eligibility or frequency of screening. Furthermore, risk models such as Shin *et al*,^25^ which separate risk by subsite, could be used to target colonoscopy rather than sigmoidoscopy in screening programmes that use this modality. Overall, our results show that several published risk models are good candidates for further evaluation in impact studies and have potential utility in the clinical and screening settings.

10.1136/gutjnl-2017-315730.supp3 Supplementary file 3 Supplementary Figure 2: Calibration plots of colorectal cancer risk models within the UK Biobank. Time horizon was 5 years for all models.

10.1136/gutjnl-2017-315730.supp4 Supplementary file 4 Supplementary Figure 3: Calibration plots of colorectal cancer risk models within the European Prospective Investigation into Cancer and Nutrition. Time horizon was 10 years for all models except Steffen *et al*^10^ which was 5 years.

10.1136/gutjnl-2017-315730.supp5 Supplementary file 5 Supplementary Figure 4: Calibration plot of colon cancer risk models within the UK Biobank. Time horizon was 5 years.

10.1136/gutjnl-2017-315730.supp6 Supplementary file 6 Supplementary Figure 5: Calibration plots of colon cancer risk models within the European Prospective Investigation into Cancer and Nutrition. Time horizon was 10 years for Ma *et al*^9^ and 5 years for Steffen *et al.*^10^

10.1136/gutjnl-2017-315730.supp7 Supplementary file 7 Supplementary Figure 6: Calibration plot of rectal cancer risk models within the UK Biobank. Time horizon was 5 years.

10.1136/gutjnl-2017-315730.supp8 Supplementary file 8 Supplementary Figure 7: Calibration plots of rectal cancer risk models within the European Prospective Investigation into Cancer and Nutrition. Time horizon was 10 years for Ma *et al*^9^ and 5 years for Steffen *et al.*^10^
