# Supplementary material for: Comparison of prognostic models to predict the occurrence of colorectal cancer in asymptomatic individuals: a systematic literature review and external validation in the EPIC and UK Biobank prospective cohort studies
Source: Gut. 2018 Apr 3;68(4):672–83. doi: 10.1136/gutjnl-2017-315730 (PMC6580880; doi:10.1136/gutjnl-2017-315730)
Supplement: Supplementary file 1 [file gutjnl-2017-315730supp001.pdf]

Supplementary Table 1: Comparison of the covariates and factors in the published models with their reconstruction in the external validation

Variable	Authors	Covariate or baseline factor for population incidence calculation	Covariate or baseline factor for population incidence calculation in the UK Biobank	Covariate or baseline factor for population incidence calculation in EPIC
Age	All	Age on entering the risk period	Variable available	Variable available
Ethnicity	Freedman et al [1]	Ethnicity of the participant.<br>The original model was for White participants. However the published code [2] also provided tailoring of the model to African-Americans, Hispanics and Asian-Americans.	Variable available.<br>Due to the predominance of White participants and the tailoring of the model to the USA population, the model was validated only in the White population.	Variable not available.<br>No suitable surrogates available. Set all to White.
	Wells et al [3]	Ethnicity of the participant.<br>Categorical variable: (i) Latino, (ii) Hawaiian, (iii) White, (iv) Black and (v) Japanese	Variable available.<br>Due to the predominance of White participants and the tailoring of the model to the USA population, the model was validated only in the White population.	Variable not available.<br>No suitable surrogates available. Set all to White.
Education	Wells et al [3]	Years of education.<br>Continuous variable.<br>The online version of this model had a maximum value of 20 years and so this cap was used in the validation.[4]	Variable not available.<br>Constructed by deducting school starting age from age at completing full time education. If this resulted in a negative value it was reset to zero.	Variable not available.<br>Constructed by deducting country specific school starting ages from the age at leaving school. If this resulted in a negative value it was reset to zero.
Height	Colditz et al [5]	Height<br>Dichotomous variable (threshold 5 feet 7 inches or taller for women and 5 feet 10 inches or taller for men)	Variable available.	Variable available.<br>The adjusted value of height was used which incorporated measured heights in EPIC along with the addition of calibrated self-reported heights from the Oxford Health Conscious Group. [6]
	Shin et al [7]	Height<br>Categorical variable: (i) ≤151cm, (ii) > 151 to ≤ 155cm, (iii) >155 to ≤ 158cm, (iv) >158cm.	Variable available.	Variable available.<br>The adjusted value of height was used which incorporated measured heights in EPIC along with the addition of calibrated self-reported heights from the Oxford Health Conscious Group.[6]
Body mass index	Colditz et al [5]<br>Driver et al [8]<br>Freedman et al [1]<br>Ma et al [9]<br>Shin et al [7]<br>Steffen et al [10]<br>Wells et al [3]	Body Mass Index (kilograms per meter squared)<br>Continuous or categorical variable.	Variable available	Variable available<br>The adjusted value of body mass index was used. This was calculated from measured heights and weights (adjusted for clothing) with the addition of calibrated self-reported heights and weights from the Oxford Health Conscious Group.[6]

Family History	Colditz et al [5]	Family History (parent or sibling) of colon cancer.<br>Dichotomous variable (presence or absence)	Variable not available<br>Constructed by incorporating history of "bowel cancer" in (i) father, (ii) mother and (iii) siblings. It was not possible to differentiate between colorectal and colon cancer, therefore all cases were assumed to be colon cancer.	Variable not available.<br>As the variables for the construction of a surrogate were not widely available and participant numbers were further constrained by the requirement for complete observations across the model's variables it was set to 0 for all participants.
	Freedman et al [1]	Family History (first degree relative) of colorectal cancer.<br>Categorical variable: (i) 0 relatives, (ii) 1 relative and (iii) 2 or more relatives.	Variable not available.<br>Constructed by incorporating history of "bowel cancer" in (i) father, (ii) mother and (iii) siblings. No available data for history of "bowel cancer" in a participant's offspring.	Variable not available.<br>As the variables for its construction were not widely available and participant numbers were further constrained by the requirement for complete observations across the model's variables it was set to 0 for all participants.
	Shin et al [7]	Family History of cancer<br>Dichotomous variable (Presence or absence)	Variable not available.<br>Constructed based on family history of "bowel cancer", "breast cancer", "lung cancer" and "prostate cancer" in (i) father, (ii) mother and (iii) siblings. No available data for history of cancer in a participant's offspring.	Variable not available.<br>Set to zero for all participants.
	Taylor et al [11]	Family History (first through third degree relatives) of colorectal cancer.<br>Categorical variable with multiple categories.	Variable not available.<br>Constructed by incorporating history of "bowel cancer" in (i) father, (ii) mother and (iii) siblings. No available data for history of "bowel cancer" in a participant's offspring. As the cohort only contained first degree relatives the familial relative risk for this degree of resolution was taken from a previously published paper.[12]	Variable not available.<br>However as, aside from age, this was the only variable included in the model it was reconstructed since the numbers eligible were not further constrained by the availability of data for other variables. It was constructed by incorporating history of colorectal cancer in (i) father, (ii) mother and (iii) siblings. There was no available data for history of colorectal cancer in a participant's offspring. As the model only contained first degree relatives the familial relative risk for this degree of resolution was taken from a previously published paper.[12]
	Wells et al [3]	Family History (first degree relative) of colon cancer<br>Dichotomous variable (presence or absence)	Variable not available.<br>Constructed by incorporating history of "bowel cancer" in (i) father, (ii) mother and (iii) siblings. It was not possible to differentiate between colorectal and colon cancer, therefore all cases were assumed to be colon cancer. No available data for history of "bowel cancer" in a participant's offspring.	Variable not available.<br>As the variables for the construction of a surrogate were not widely available and participant numbers were further constrained by the requirement for complete observations across the model's variables it was set to 0 for all participants.
Diabetes	Steffen et al [10]	History of Diabetes<br>Dichotomous variable (presence or absence)	Variable available	Variable available
	Wells et al [3]	History of Diabetes<br>Dichotomous variable (presence or absence)	Variable available	Variable available

Endoscopy / Screening	Colditz et al [5]	History of faecal occult blood testing or sigmoidoscopy in the preceding 10 years beginning at age 50.<br>Dichotomous variable (presence or absence)	Variable not available<br>Utilised similar variables which detailed prior bowel cancer screening (faecal occult blood tests or endoscopy) as well as the time since the last screen. This differed from the original variable construction by the inclusion of colonoscopy investigations in addition to sigmoidoscopy. The 50 year age threshold was not applied as this was not a limitation in our data.	Variable not available.<br>No suitable surrogates available so set to absent for all participants.
	Freedman et al [1]	Endoscopy history in the preceding 10 years and the presence of polyps.<br>Categorical variable:<br>(i) Endoscopy and no history of polyps in the last 10 years.<br>(ii) No endoscopy in the last 10 years.<br>(iii) Endoscopy and history of polyps in the last 10 years.<br>(iv) Endoscopy and polyps unknown.	Variable not available.<br>Constructed from variables detailing prior bowel cancer screening (faecal occult blood tests or endoscopy), time since the last screen and the presence of colon or rectal adenoma/polyps. This differed from the original variable construction as (i) it includes faecal occult blood tests, therefore widening the remit and (ii) there was no indication of when the polyp (if present) was diagnosed. As such the 10 year threshold was changed to "ever", again widening the remit of the model.	Variable not available.<br>Constructed from variables that detailed the presence of large bowel polyps and the age at which they were identified. It was inferred that those with polyps diagnosed within the preceding 10 years had undergone endoscopy and so were placed in category (iii), while those with polyps diagnosed more than 10 years prior would have undergone further surveillance and so were placed in category (i). All others were placed in category (iv).
	Steffen et al [10]	Ever had colorectal cancer screening<br>Dichotomous variable (presence or absence)	Variable available.	Variable not available.<br>No suitable surrogates were available so set to absent for all participants.
Inflammatory Bowel Disease	Colditz et al [5]	Inflammatory Bowel Disease for 10 or more years	Variable not available<br>It was constructed by utilising self-reported data for diagnosis but without quantification of the duration.	Variable not available.<br>No suitable surrogates available so set to absent for all participants.
Menopause/ oestrogen use	Colditz et al [5]	Use of birth control pills for 5 years or more<br>Dichotomous variable (presence or absence)	Variable not available<br>Constructed from variables detailing (i) ever use of the oral contraceptive pill (OCP), (ii) age at starting the OCP and (iii) age at which the OCP was last used.	Variable not available.<br>Constructed from variables detailing length of OCP use and ever use of the OCP.
	Colditz et al [5]	Use of postmenopausal hormones for 5 years or more<br>Dichotomous variable (presence or absence)	Variable not available<br>Constructed from variables detailing (i) ever use of hormone replacement therapy (HRT), (ii) age at starting HRT and (iii) age at which HRT was last used.	Variable not available.<br>Constructed from variables detailing length of HRT use and ever use of HRT.
	Freedman et al [1]	Oestrogen status in the preceding two years. Assessed by menopause status and the use of HRT.<br>Dichotomous variable (positive or negative).	Variable not available.<br>Constructed using (i) age at menopause, (ii) history of oophorectomy, (iii) age at oophorectomy, (iv) HRT ever use, (v) age at starting HRT and (vi) age at last use of HRT.	Variable not available.<br>Constructed using (i) age at menopause, (ii) history of oophorectomy, (iii) age at oophorectomy, (iv) HRT ever use, (v) age at starting HRT and (vi) duration of HRT use
	Wells et al [3]	Oestrogen use. Defined as "female hormones administered by pill, injection or patch for menopause or other reasons" [3].<br>Categorical variable: (i) current use, (ii) prior use, (iii) never use.	Variable not available.<br>Constructed from (i) ever use of the OCP, (ii) age last used OCP, (iii) ever use of HRT and (iv) age last used HRT.	Variable not available<br>Constructed from (i) ever use of the OCP, (ii) current use of the OCP, (iii) ever use of HRT and (iv) current use of HRT.

Non-steroidal anti-inflammatory drugs (NSAIDs)	Colditz et al [5]	Daily aspirin use for 15 years or more.<br>Dichotomous variable (presence or absence)	Variable not available.<br>It was constructed by utilising a variable that detailed the regular use of aspirin but without further quantification of the frequency or duration of use.	Variable not available.<br>A variable was present which identified regular users of NSAIDs but without further quantification of the type, frequency or duration of use. Furthermore it was only available in a limited number of individuals. As such it was set to nil for all participants.
	Freedman et al [1]	In the original model this was classified as use of aspirin and other NSAIDs for at least three times a week, for a minimum of 1 month, in the preceding year. However the published script for men separated this into aspirin and non-aspirin containing NSAIDs.[2]<br>Dichotomous variable (presence or absence)	Variable not available.<br>It was constructed by utilising variables that detailed the regular use of aspirin and ibuprofen but without further quantification of the frequency or duration of use. In the female model the two variables subtypes were combined while in the male model they were entered as separate variables.	Variable not available.<br>A variable was present which identified regular users of NSAIDs but without further quantification of the type, frequency or duration of use. Furthermore it was only available in a limited number of individuals. As such it was set to nil for all participants (including in the male model where NSAIDs were subdivided).
	Wells et al [3]	Use of Aspirin (men) or NSAIDs (women) twice per week.<br>Categorical variable: (i) yes, currently, (ii) yes, not currently and (iii) no.	Variable not available.<br>It was constructed by utilising variables that detailed the regular use of aspirin and ibuprofen but without further quantification of the frequency or duration of use.	Variable not available.<br>A variable was present which identified regular users of NSAIDs but without further quantification of the type, frequency or duration of use. Furthermore it was only available in a limited number of individuals. As such it was set to nil for all participants.
Physical activity	Colditz et al [5]	In its validation [13] physical activity was defined in metabolic equivalent tasks (MET).<br>Dichotomous variable (threshold 25 MET hours per week or greater).	Variable not available.<br>Constructed from variables detailing the number of days per week the participant undertook moderate or vigorous physical activity and the usual number of minutes spent doing so. This was converted into a total MET score (hours per week) following the International Physical Activity Questionnaire Guidelines.[14]	Variable available<br>(recreational and household activities)
	Freedman et al [1]	Current leisure-time vigorous activity.<br>Categorical variable: (i) 0 hours per week, (ii) 0-2 hours per week, (iii) 2-4 hours per week, (iv) 4+ hours per week.	Variable not available.<br>Constructed from variables detailing the number of days per weeks the participant undertook vigorous physical activity as well as the usual number of minutes spent undertaking it on a typical day. This was converted into the number of hours per week following the International Physical Activity Questionnaire Guidelines.[14]	Variable available<br>(MET hours per week from vigorous recreational physical activity were converted into hours per week)
	Ma et al [9]	MET hours per day were calculated based on time spent at: (i) heavy physical work or strenuous activity, (ii) walking or standing, (iii) sedentary and (iv) asleep or others.<br>Continuous variable.	Variable not available.<br>Constructed from variables detailing the number of days per week and duration the participant walked, or undertook moderate or vigorous physical activity (following the International Physical Activity Questionnaire Guidelines [14]) as well as the duration of sleep per day. Walking was the sole contributor to the walking or standing variable, as was sleep to the asleep or others variable. Moderate and vigorous physical activity were summed to create the heavy physical work / strenuous activity variable. Sedentary was then derived from the remaining time in the day. These were then all	Variable not available.<br>Constructed from variables which provided MET hours per week from walking, cycling, gardening, sport, housework, DIY and stair climbing after converting them into hours of activity per day. All but walking were summed to create the heavy physical work/strenuous activity variable, while walking was the sole contributor to the walking or standing variable. The remaining time was then classified as asleep or others unless it exceeded 8 hours in which case the excess time contributed to the sedentary category. These were then all converted into METs using the authors' conversion factors and summed.

			converted into METs using the authors' conversion factors and summed.	
	Wells et al [3]	Hours of moderate physical activity per day.<br>However the description of the type of physical activity used within their derivation cohort was "moderate or strenuous activity per day" and so this definition was utilised.<br>The online version of this model had a maximum value of 4 hours and so this cap was used in the validation.[4]<br>Continuous variable.	Variable not available.<br>Constructed from variables detailing the number of days per weeks the participant undertook moderate or vigorous physical activity and the usual duration. This was summed to calculate total number of hours per day following the International Physical Activity Questionnaire Guidelines.[14]	Variable not available.<br>Constructed by converting variables detailing MET hours per week from walking, cycling, gardening, sport, housework, DIY and stair climbing into hours per day and summing.
Smoking	Driver et al [8]	Smoking Status<br>Dichotomous variable (never smoker or current/previous smoker)	Variable available.	Variable available.
	Freedman et al [1]	Years of cigarette smoking<br>Categorical variable: (i) 0, (ii) >0 to <15, (iii) ≥15 to <35 and (iv) ≥35	Variable not available.<br>Constructed from variables detailing (i) age started cigarette smoking, (ii) age stopped cigarette smoking and (iii) periods of stopping smoking.	Variables available.
	Freedman et al [1]	Usual number of cigarettes smoked per day for current and former smokers.<br>Categorical variable: (i) never, (ii) >0 to <11, (iii) ≥11 to ≤20 and (iv) >20.	Variable not available.<br>Constructed from variables available detailing number of cigarettes currently or previously smoked a day.	Variables available.
	Ma et al [9]	Smoking Status<br>Categorical variable: (i) Never smokers, (ii) current smokers (iii) former smokers.	Variable available.	Variable available.
	Steffen et al [10]	Smoking Status<br>Categorical variable: (i) Never smokers, (ii) current smokers (iii) former smokers.	Variable available.	Variable available.
	Wells et al [3]	Pack years of smoking.<br>The online version of this model had a maximum value of 50 pack years and so this cap was used in the validation [4]<br>Continuous variable.	Variable not available.<br>Constructed using a purpose built algorithm that incorporated variables detailing number of cigarettes smoked, duration of smoking and periods of smoking cessation. This was based on the pack year calculation developed for the UK Biobank data by Professor Martin Tobin, University of Leicester.[15]	Variable not available.<br>Constructed from variables detailing lifetime average number of cigarettes smoked and duration of smoking.
Alcohol	Colditz et al [5]	Frequency of alcohol consumption<br>(more than seven servings per week)<br>Dichotomous variable (presence or absence)	Variable available<br>(the category of "daily or almost daily" was regarded to equate to more than seven servings per week).	Variable not available<br>Constructed from a surrogate, detailing grams of alcohol per day, after conversion to drinks per day (12 grams of alcohol per drink).
	Driver et al [8]	Frequency of alcohol consumption<br>(once a week or more)<br>Dichotomous variable (presence or absence)	Variable available<br>(the category of "once or twice a week" was regarded to equate to once a week or more).	Variable not available<br>Constructed from a surrogate, detailing grams of alcohol per day, after conversion to drinks per week (12 grams of alcohol per drink).

	Ma et al [9]	Alcohol consumption<br>Categorical variable: (i): never, (ii) occasional, (iii) < 300 grams per week, (iv) ≥300 grams per week.	Variable not available.<br>Constructed from variables detailing (i) the frequency of alcohol consumption as a whole as well as (ii) the frequency and volume of different types of alcoholic drinks. Typical values for units of alcohol in different drinks as well as the number of grams of alcohol per unit (8g) were obtained from a UK Government publication [16].	Variable not available<br>Constructed from variables detailing frequency of alcohol consumption and grams of alcohol per day.
	Shin et al [7]	Alcohol consumption<br>Categorical variable: (i) 0 g/day, (ii) 1-14.9 g/day, (iii) 15-24.9 g/day, (iv) 25 or more g/day.	Variable not available.<br>Constructed from variables detailing the frequency and volume of different types of alcoholic drinks. Typical values for units of alcohol in different drinks as well as the number of grams of alcohol per unit (8g) were obtained from a UK Government publication.[16]	Variable available.
	Steffen et al [10]	Drinks per day<br>Continuous variable	Variable not available.<br>Constructed from variables documenting the frequency of drinking different types of alcoholic drinks.	Variable not available<br>Constructed from a surrogate, detailing grams of alcohol per day, after conversion to drinks per day (12 grams of alcohol per drink).
	Wells et al [3]	Drinks per day<br>Continuous variable<br>The online version of this model had a maximum value of 12 drinks and so this cap was used in the validation.[4]	Variable not available.<br>Constructed from variables documenting the frequency of drinking different types of alcoholic drinks.	Variable not available<br>Constructed from a surrogate, detailing grams of alcohol per day, after conversion to drinks per day (12 grams of alcohol per drink).
Red Meat/Meat	Colditz et al [5]	Eating 1.2 servings or more of red meat a day<br>Updated definition and relative risk were taken from a subsequent validation paper. [13]<br>Dichotomous variable (presence or absence)	Variable not available<br>Constructed by merging beef, lamb/mutton, pork and processed meat intake. Incorporated processed meat due to the fact that red meat would be a substantial constituent of it.	Variable not available<br>Constructed by merging red and processed meat (due to the fact that red meat would be a substantial constituent of processed meat). A serving was taken to be 113g.
	Shin et al [7]	Meat consumption per week<br>Categorical variable: (i) ≤1 time, (ii) 2-3 times, (iii) ≥ 4 times.	Variable not available.<br>Constructed by merging beef, lamb/mutton, pork, poultry and processed meat intake.	Variable not available.<br>Variable detailing the total weight of meat intake per day was extrapolated to per week and converted to servings (113g was taken to one serving).
	Wells et al [3]	Ounces of red meat intake per day<br>The online version of this model had a maximum value of 5 ounces and so this cap was used in the validation.[4]<br>Continuous variable.	Variable not available<br>Constructed by merging beef, lamb/mutton, pork and processed meat intake. Incorporated processed meet due to the fact that red meat would be a substantial constituent of it.	Variable not available<br>Constructed by merging red and processed meat (due to the fact that red meat would be a substantial constituent of processed meat). Converted to ounces.
Vegetables	Colditz et al [5]	Eating 3 or more servings of vegetables per day<br>Dichotomous variable (presence or absence)	Variable not available<br>Constructed by summing two surrogates detailing tablespoons of raw and cooked vegetable eaten per day.	Variable not available.<br>Constructed from a surrogate, detailing grams of vegetables eaten per day.
	Freedman et al [1]	Eating 5 or more servings of vegetables per day<br>Dichotomous variable (presence or absence)	Variable not available<br>Constructed by summing two surrogates detailing tablespoons of raw and cooked vegetable eaten per day.	Variable not available.<br>Constructed from a surrogate, detailing grams of vegetables eaten per day.

Multivitamins	Colditz et al [5]	Regular multivitamin use<br>Dichotomous variable (presence or absence)	Variable available	Variable not available<br>"Have you taken vitamins/minerals" was used as a surrogate
	Wells et al [3]	Regular multivitamin use<br>Dichotomous variable (presence or absence)	Variable available	Variable not available<br>"Have you taken vitamins/minerals" was used as a surrogate

Supplementary Table 2: General characteristics of the populations used for the original model derivation, The European Prospective Investigation into Cancer and Nutrition (EPIC) validation and UK Biobank validation

Model	Population Origin	Country	Characteristics / Eligibility Criteria	Mean Age (or as described)	Mean BMI (kg/m^2) (or as described)	Participants (cases)	Outcome Cancer type
**Derivation Cohort**							
Colditz [5]	Model was constructed through expert opinion, and did not utilise a specific cohort						
Driver [8]	Physician's Health Study. Followed from 1982 to 2004.	USA	Male aged 40-84<br>Apparently healthy. No history of cardiovascular disease or other serious illnesses, no history of cancer (bar non-melanoma skin cancer) and no indication or contraindication to analgesia usage.	40-49 (41.1%)<br>50-59 (33.9%)<br>60-69 (18.5%)<br>≥70 (6.5%)	<25 (57.6%)<br>25-29.9 (38.2%)<br>≥30 (4.2%)	21,581 (485)	Colorectal
Freedman [1]	Case-control<br>1991 to 1994	USA	Male and Female.<br>Cases excluded if prior colorectal cancer diagnosis, familial adenomatous polyposis, ulcerative colitis or Crohn's documented in the pathology report. Controls matched by sex and age (5 year age group)	50-59: Case = 319, Control = 347<br>60-69: Case = 606, Control = 751<br>≥70: Case = 671, Control = 876	Male:<br>≤24.9: Case = 220, Control = 337<br>25-≤30: Case = 426, Control = 514<br>>30: Case = 245, Control = 207<br><br>Female (Distal Colon Cases only)<br>≤29.9: Case= 246, Control = 726<br>≥30: Case= 88, Control = 190	1,599 cases<br>1,974 controls	Colon
	Case-control<br>1997 to 2002	USA	Male and Female<br>Cases excluded if prior colorectal cancer diagnosis, familial adenomatous polyposis, ulcerative colitis or Crohn's documented in the pathology report. Controls matched by sex and age (5 year age group)	50-59: Case = 175, Control = 225<br>60-69: Case = 261, Control = 316<br>≥70: Case = 228, Control = 318	Female:<br>≤29.9: Case= 192, Control =298<br>≥30: Case= 75, Control =83	664 cases<br>859 controls	Rectal
Ma [9]	Japan Public Health Center-based Prospective Study. Cohort II. Followed from 1993 to 2005.	Japan	Male aged 40 – 69<br>Excluded if a history of cardiovascular disease or cancer.	52.9 (S.D. 8.8)	23.4 (S.D. 2.9)	28,115 (543)	Colorectal
Shin [7]	Korean National Health System. Followed from 1996-1997 to 2004-2005	Korea	Male and Female aged 30 - 80<br>Participated in a National Health Insurance Corporation medical examination.	Not available	Not available	1,326,058 (9,147)	Colorectal

			Excluded if prior cancer diagnosis or missing variables				
Steffen [10]	45 and Up Study. Followed from 2006-2008 to 2011	Australia	Male and Female aged 45 or older<br>Excluded if prevalent cancer (bar non-melanoma skin cancer), missing study entry date, body mass index <15 or >50 kg/m^2 or extreme values for activity or food intake.	61.2 (S.D. 10.8)	27.0 (S.D. 4.9)	197,874 (1,103)	Colorectal
Taylor [11]	Utah Population Database (UPDB).	USA	Male and Female<br>Familial relative risk was calculated from the descendants of original Utah pioneers with ≥3 generations of genealogy data.[12] The CRC morbidity rates and all-cause mortality rates were calculated from UPDB data (1981 to 1985).[11]			2,327,327 (94,931)	Colorectal
Wells [3]	Multiethnic Cohort Study. Followed from 1993-1996 to 2004	USA	Male and Female aged over 45.<br>Excluded if a prior diagnosis of colorectal cancer or adenomatous polyps	Male<br>Case = 64.2 (S.D. 7.8)<br>Non-case = 59.8 (S.D. 8.9)<br><br>Female<br>Case = 64 (S.D. 7.9)<br>Non-case = 59.5 (S.D. 8.8)	Male<br>Case = 26.6 (S.D. 4.2)<br>Non-case = 26.6 (S.D. 4.1)<br><br>Female<br>Case = 26.6 (S.D. 5.7)<br>Non-case = 26.4 (S.D. 5.5)	180,630 (2,762)	Colorectal
**Validation Cohorts**							
	UK Biobank †. Followed 2006-2010 to 2015.	Great Britain	Male and Female aged 37 – 73<br>Excluded if prior cancer diagnosis (bar non-melanoma skin cancer), zero or negative follow-up. Additionally new diagnosis of appendiceal cancers were excluded with the participants being censored at diagnosis.	56.3 (S.D. 8.1)	27.4 (S.D. 4.8)	475,629 (2,617)	Colorectal
	European Prospective Investigation into Cancer and Nutrition †. Followed 1991-2001 to 2013	Europe	Male and Female aged 17 – 98<br>Excluded if prior cancer diagnosis, zero length follow-up, missing the date of cancer diagnosis, missing the data of vital status check-up.<br>Additionally new diagnosis of appendiceal cancers were excluded with the participants being censored at diagnosis.	51.3 (S.D. 10.0)	25.8 (S.D. 4.3)‡	491,992 (6,467)	Colorectal

† The description of the validation cohorts is as they were available for use with all models after eligibility criteria had been applied. Participants available for validation of specific models varied depending on the variables required. ‡ Variable available for 400,746 participants.

Supplementary Table 3: Comparison of eligible and ineligible male participants in the UK Biobank

	UKB Male	Colditz [5]		Driver [8]		Freedman [1]		Ma [9]		Shin [7]		Steffen [10] β		Taylor [11] β		Wells [3]	
		Eligible	Ineligible	Eligible	Ineligible	Eligible	Ineligible	Eligible	Ineligible	Eligible	Ineligible	Eligible	Ineligible	Eligible	Ineligible	Eligible	Ineligible
Number of Participants	219,385	157,638	61,747	216,440	2,945	118,439	100,946	196,524	22,861	145,723	73,662	185,914	33,471	177,031	42,354	93,608	125,777
(% of cohort)		(71.9)	(28.1)	(98.7)	(1.3)	(54.0)	(46.0)	(89.6)	(10.4)	(66.4)	(33.6)	(84.7)	(15.3)	(80.7)	(19.3)	(42.7)	(57.3)
Colorectal Cases■	1,258			1,236	22	823	435	1,102	156			1,085	173	990	268	559	699
(C.I.R. /100,000 years)	(118.9)			(118.4)	(157.3)	(144.8)	(88.9)	(116.2)	(142.6)			(121.2)	(106.7)	(115.8)	(132.0)	(123.9)	(115.3)
Colon	768	518	250					670	98	229 Ω	131 Ω	651	117				
(C.I.R. /100,000 years)	(72.6)	(68.0)	(84.4)					(70.7)	(89.6)	(32.5)	(37.0)	(72.7)	(72.2)				
Rectal	482							424	58			428	54				
(C.I.R. /100,000 years)	(45.6)							(44.7)	(53.0)			(47.8)	(33.3)				
Follow-up (years)	1,057,724	761,536	296,188	1,043,740	13,983	568,480	489,244	948,335	109,388	703,758	353,966	895,575	162,148	854,696	203,028	451,300	606,424
Age^																	
% with no data	0.0	0.0	0.0	0.0	0.0	0.0	0.0	0.0	0.0	0.0	0.0	0.0	0.0	0.0	0.0	0.0	0.0
Of those with data	56.5	56.2	57.5	56.5	56.8	60.0	52.5	56.4	57.4	56.5	56.7	56.7	55.8	56.3	57.6	56.9	56.3
(S.D.)	(8.2)	(8.2)	(8.2)	(8.2)	(8.5)	(5.4)	(9.0)	(8.2)	(8.3)	(8.1)	(8.4)	(8.2)	(8.4)	(8.2)	(8.2)	(8.1)	(8.3)
BMI kg/m^2^																	
% with no data	0.7	0.0	2.6	0.0	54.0	0.0	1.6	0.0	7.0	0.0	2.2	0.0	4.8	0.6	1.4	0.0	1.3
Of those with data	27.8	27.7	28.3	27.8	28.4	27.8	27.9	27.8	28.5	27.6	28.3	27.7	28.4	27.7	28.3	28.1	27.6
(S.D.)	(4.2)	(4.1)	(4.5)	(4.2)	(4.7)	(4.1)	(4.4)	(4.2)	(4.7)	(4.0)	(4.6)	(4.1)	(4.8)	(4.2)	(4.5)	(4.2)	(4.3)
Smoking status																	
% with no data	0.6	0.2	1.7	0.0	46.3	0.0	1.4	0.0	6.0	0.2	1.4	0.0	4.1	0.3	2.0	0.0	1.1
Of those with data																	
% Never	49.2	51.1	44.3	49.2	46.1	47.9	50.7	49.8	43.4	49.4	48.7	48.9	51.1	50.5	43.5	46.4	51.3
% Previous	38.2	37.7	39.4	38.2	31.7	42.1	33.5	38.2	37.5	39.2	36.0	39.1	32.7	37.7	40.1	40.8	36.2
% Current	12.7	11.3	16.4	12.6	22.1	10.1	15.8	12.0	19.1	11.3	15.4	12.1	16.2	11.8	16.4	12.8	12.6
Alcohol status																	
% with no data	0.3	0.0	1.2	0.0	25.1	0.0	0.7	0.0	3.2	0.0	1.0	0.0	2.2	0.1	1.5	0.0	0.6
Of those with data																	
% Never	2.8	2.2	4.5	2.8	8.9	1.6	4.3	2.5	6.0	2.9	2.7	3.1	1.5	2.5	4.2	1.6	3.8
% Previous	3.5	3.0	4.9	3.5	7.0	2.9	4.2	3.3	5.4	3.7	3.1	3.9	1.4	3.2	4.9	3.1	3.8
% Current	93.7	94.8	90.6	93.8	84.1	95.5	91.5	94.2	88.6	93.4	94.2	93.1	97.1	94.3	90.9	95.3	92.5
Days/week of vigorous physical activity ^																	
% with no data	4.8	0.0	17.0	4.4	31.6	0.0	10.4	0.0	45.9	2.7	8.9	3.7	10.6	3.4	10.7	0.0	8.3
Of those with data	2.1	2.1	2.0	2.1	2.0	2.0	2.2	2.1	1.9	2.1	2.0	2.1	2.0	2.1	2.1	2.1	2.0
(S.D.)	(2.1)	(2.0)	(2.1)	(2.0)	(2.3)	(2.0)	(2.1)	(2.0)	(2.3)	(2.0)	(2.1)	(2.0)	(2.1)	(2.0)	(2.2)	(2.1)	(2.0)
Processed meat consumption																	
% with no data	0.5	0.0	1.8	0.2	19.8	0.0	1.0	0.1	3.9	0.0	1.5	0.2	2.4	0.1	2.0	0.0	0.9
Of those with data																	
% Never	5.4	5.4	5.6	5.4	9.4	4.6	6.5	5.4	6.1	5.2	6.0	5.3	6.2	5.4	5.5	3.8	6.7
% < 1 / week	21.4	21.6	20.7	21.4	20.0	22.0	20.6	21.5	20.1	21.0	22.1	21.1	22.7	21.5	20.9	20.0	22.4
% 1 / week	29.8	30.1	29.0	29.8	28.2	30.8	28.6	29.9	29.3	30.3	28.8	30.0	28.9	30.0	28.8	30.5	29.3
% 2-4 / week	36.9	36.7	37.4	36.9	34.7	36.6	37.2	36.9	37.1	37.3	36.0	37.2	35.2	36.8	37.4	39.1	35.2
% 5-6 / week	5.2	5.0	5.6	5.2	5.7	4.8	5.6	5.1	5.6	5.0	5.4	5.1	5.4	5.0	5.7	5.3	5.1
% ≥ 1 / day	1.4	1.2	1.7	1.4	2.0	1.1	1.6	1.3	2.0	1.2	1.7	1.3	1.7	1.3	1.7	1.3	1.4

The UK Biobank (UKB) was assessed over a 5 year time horizon from recruitment. β These models were not sex-specific and so both male and female participants were eligible. ■ In the UKB some colorectal cancer diagnosis included more than one anatomical site. If the cancer encompassed both colon and rectum at the date of first diagnosis it was classified as colorectal and was not included in the subsite analysis (e.g. colon or rectal). ^ Mean values with standard deviation (S.D.). Ω Right colon cancer only. Cancer site was defined by the International Statistical Classification of Disease and Related Health Problems, 10th Revision,[17] colorectal encompassed C18-C20, colon C18 and rectal C19 and C20.

Supplementary Table 4: Comparison of eligible and ineligible male participants in The European Prospective Investigation into Cancer and Nutrition

	EPIC Male	Colditz [5]		Driver [8]		Freedman [1]		Ma [9]		Shin [7]		Steffen [10 β]		Taylor [11 β]		Wells [3]	
		Eligible	Ineligible	Eligible	Ineligible	Eligible	Ineligible	Eligible	Ineligible	Eligible	Ineligible	Eligible	Ineligible	Eligible	Ineligible	Eligible	Ineligible
Number of Participants	148,007	93,863	54,144	122,650	25,357	56,290	91,717	110,784	37,223	124,293	23,714	111,667	36,340	25,273	122,734	41,587	106,420
(% of cohort)		(63.4)	(36.6)	(82.9)	(17.1)	(38.0)	(62.0)	(74.9)	(25.1)	(84.0)	(16.0)	(75.4)	(24.6)	(17.1)	(82.9)	(28.1)	(71.9)
Colorectal Cases	1,472			1,278	194	771	701	1,191	281			463	179	261	1,211	395	1,077
(C.I.R. /100,000 years)	(107.0)			(111.9)	(83.5)	(149.2)	(81.8)	(115.7)	(81.6)			(85.2)	(101.8)	(108.3)	(106.9)	(101.5)	(109.3)
Colon	855	607	248					676	179	115 Ω	30 Ω	258	117				
(C.I.R. /100,000 years)	(62.2)	(69.1)	(50.0)					(65.7)	(52.0)	(19.0)	(26.2)	(47.5)	(66.6)				
Rectal	617							515	102			205	62				
(C.I.R. /100,000 years)	(44.9)							(50.0)	(29.6)			(37.7)	(35.3)				
Follow-up time (years)	1,374,039	878,437	495,602	1,141,586	232,453	516,656	857,383	1,029,610	344,430	605,178	114,315	543,701	175,792	241, 050	1,132,990	389,014	985,025
Age^																	
% with no data	0.0	0.0	0.0	0.0	0.0	0.0	0.0	0.0	0.0	0.0	0.0	0.0	0.0	0.0	0.0	0.0	0.0
Of those with data	52.3	54.2	49.0	53.7	45.3	57.5	49.1	54.0	47.2	53.7	44.6	53.4	48.7	53.3	52.0	51.8	52.5
(S.D.)	(10.2)	(7.0)	(13.4)	(7.3)	(17.0)	(4.9)	(11.2)	(7.2)	(14.9)	(7.3)	(17.3)	(7.2)	(15.6)	(7.8)	(10.6)	(7.5)	(11.1)
BMI kg/m^2^																	
% with no data	2.3	0.0	6.2	0.0	13.3	0.0	3.7	0.0	9.0	0.0	14.2	0.0	9.3	6.8	1.3	0.0	3.2
Of those with data	26.5	26.7	26.2	26.7	25.4	27.0	26.2	26.8	25.5	26.7	25.3	26.8	25.6	27.6	26.3	27.0	26.3
(S.D.)	(3.7)	(3.6)	(3.8)	(3.6)	(3.7)	(3.6)	(3.7)	(3.6)	(3.7)	(3.6)	(3.7)	(3.7)	(3.6)	(3.5)	(3.7)	(3.6)	(3.7)
Smoking status																	
% with no data	2.0	0.4	4.6	0.0	11.5	0.0	3.2	0.0	7.8	1.3	5.3	0.0	8.0	3.3	1.7	0.0	2.7
Of those with data:																	
% Never	33.3	29.7	39.8	31.2	44.6	33.8	33.0	29.7	44.8	31.2	44.6	31.1	40.8	31.2	33.7	34.2	32.9
% Previous	36.9	39.1	32.8	38.2	29.8	39.6	35.2	39.1	29.8	38.2	29.8	37.6	34.5	36.9	36.9	37.8	36.5
% Current	29.8	31.2	27.4	30.6	25.7	26.6	31.9	31.2	25.4	30.6	25.7	31.4	24.7	31.9	29.4	28.0	30.6
Alcohol status																	
% with no data	24.9	15.6	41.0	20.3	47.6	4.5	37.5	11.8	64.1	20.7	47.3	21.0	37.1	10.8	27.8	0.5	34.5
Of those with data:																	
% Never	1.6	1.3	2.4	1.5	2.5	1.5	1.8	1.5	2.5	1.5	2.5	1.5	2.2	3.2	1.3	1.8	1.5
% Previous	4.4	4.3	4.8	4.4	4.7	4.5	4.3	4.4	4.8	4.4	4.7	3.9	6.4	10.7	2.8	5.0	4.1
% Current	94.0	94.4	92.8	94.1	92.8	94.0	93.9	94.1	92.8	94.1	92.8	94.6	91.4	86.1	95.9	93.2	94.4
Vigorous recreation activity (MET hours / week) ^																	
% with no data	24.5	19.2	33.6	23.2	30.5	0.0	39.5	15.4	51.4	23.6	29.1	23.3	28.0	7.5	27.9	0.8	33.7
Of those with data	18.1	17.6	19.0	17.5	21.2	18.4	17.8	17.5	21.0	17.5	21.4	17.4	20.3	8.7	20.6	13.9	20.5
(S.D.)	(28.7)	(27.6)	(30.8)	(27.9)	(32.4)	(28.5)	(28.8)	(27.8)	(32.5)	(27.9)	(32.5)	(27.9)	(31.0)	(22.0)	(29.7)	(26.1)	(29.8)
Processed meat consumption (g/day) ^																	
% with no data	1.9	0.0	5.3	0.0	11.4	0.0	3.1	0.0	7.7	0.0	12.1	0.0	7.9	6.8	1.0	0.0	2.7
Of those with data	42.3	45.4	36.5	44.4	30.5	40.9	43.1	44.5	35.1	44.2	30.8	45.4	31.8	41.7	42.4	48.7	39.7
(S.D.)	(41.2)	(40.8)	(41.3)	(41.7)	(36.0)	(40.5)	(41.6)	(42.4)	(36.2)	(41.6)	(36.5)	(42.5)	(34.4)	(37.7)	(41.8)	(47.8)	(37.9)

The European Prospective Investigation into Cancer and Nutrition (EPIC) was assessed over a 10 year time horizon from recruitment, with the exception of Shin et al [7] and Steffen et al [10] which were assessed over 5 years. [β] These models were not sex-specific and so both male and female participants were eligible. ^ Mean values with standard deviation (S.D.). Ω Right colon cancer only. Cancer site was defined by the International Classification of Diseases for Oncology, 2nd revision (ICD-O-2),[18] colorectal encompassed C18-C20, colon C18 and rectal C19 and C20.

Supplementary Table 5: Comparison of eligible and ineligible female participants in the UK Biobank

	UKB Female	Colditz [5]		Freedman [1]		Shin [7]		Steffen [10 β]		Taylor [11 β]		Wells [3]	
		Eligible	Ineligible	Eligible	Ineligible	Eligible	Ineligible	Eligible	Ineligible	Eligible	Ineligible	Eligible	Ineligible
Number of Participants	256,244	172,807	83,437	123,991	132,253	178,665	77,579	201,704	54,540	219,484	36,760	117,367	138,877
(% of cohort)		(67.4)	(32.6)	(48.4)	(51.6)	(69.7)	(30.3)	(78.7)	(21.3)	(85.7)	(14.3)	(45.8)	(54.2)
Colorectal Cases■	892			521	371			696	196	768	124	426	466
(C.I.R. /100,000 years)	(71.7)			(86.7)	(57.7)			(71.2)	(73.5)	(72.1)	(69.6)	(74.7)	(69.1)
Colon	639	402	237					490	149				
(C.I.R. /100,000 years)	(51.4)	(47.9)	(58.6)					(50.1)	(55.9)				
Rectal	240					176	64	194	46				
(C.I.R. /100,000 years)	(19.3)					(20.3)	(17.0)	(19.8)	(17.3)				
Follow-up time (years)†	1,244,045	839,681	404,364	600,585	643,460	867,746	376,299	977,418	266,627	1,065,777	178,268	570,107	673,938
Age^													
% with no data	0.0	0.0	0.0	0.0	0.0	0.0	0.0	0.0	0.0	0.0	0.0	0.0	0.0
Of those with data	56.1	55.4	57.7	59.5	53.0	56.1	56.1	56.1	56.2	56.0	57.2	56.7	55.7
(S.D.)	(8.0)	(8.0)	(7.9)	(5.3)	8.8	(7.9)	(8.2)	(8.0)	(8.2)	(8.0)	(8.1)	(7.9)	(8.1)
BMI kg/m^2^													
% with no data	0.5	0.0	1.6	0.0	1.0	0.1	1.5	0.0	2.5	0.4	1.2	0.0	1.0
Of those with data	27.1	26.7	27.8	26.9	27.3	26.6	28.2	26.8	28.2	26.9	28.0	27.3	26.9
(S.D.)	(5.2)	(5.1)	(5.4)	(4.9)	5.4	(4.9)	(5.8)	(5.0)	(5.7)	(5.1)	(5.5)	(5.2)	(5.2)
Smoking status													
% with no data	0.6	0.2	1.3	0.2	0.9	0.3	1.1	0.0	2.6	0.3	2.1	0.0	1.0
Of those with data													
% Never	59.9	61.0	57.7	58.7	61.1	58.4	63.3	59.2	62.6	60.5	56.0	58.7	60.9
% Previous	31.1	30.9	31.6	34.0	28.4	33.2	26.4	32.3	26.7	30.9	32.3	31.7	30.6
% Current	9.0	8.2	10.8	7.3	10.6	8.5	10.2	8.5	10.7	8.6	11.7	10.0	8.5
Alcohol status													
% with no data	0.3	0.0	0.8	0.0	0.5	0.0	0.9	0.0	1.3	0.1	1.6	0.0	0.5
Of those with data													
% Never	5.9	4.8	8.2	4.4	7.3	8.0	0.9	7.1	1.4	5.5	8.6	4.9	6.7
% Previous	3.6	3.2	4.6	3.3	3.9	5.1	0.3	4.5	0.5	3.4	4.9	3.7	3.6
% Current	90.5	92.0	87.3	92.3	88.8	86.9	98.8	88.4	98.1	91.1	86.5	91.4	89.7
Days/week of vigorous physical activity ^													
% with no data	6.0	0.0	18.5	0.0	11.7	4.7	9.0	5.0	9.9	4.9	12.6	4.9	7.0
Of those with data	1.7	1.7	1.6	1.6	1.7	1.7	1.6	1.7	1.6	1.7	1.7	1.6	1.7
(S.D.)	(1.9)	1.8	(1.9)	(1.8)	(1.9)	(1.8)	(1.9)	(1.9)	(1.9)	(1.8)	(2.0)	(1.9)	(1.9)
Processed meat consumption													
% with no data ^	0.4	0.0	1.3	0.0	0.8	0.0	1.4	0.2	1.4	0.2	2.0	0.1	0.7
Of those with data													
% Never	12.6	12.9	12.0	12.0	13.3	12.6	12.8	12.6	12.7	12.6	12.7	10.3	14.7
% < 1 / week	38.0	38.6	36.9	39.8	36.4	37.8	38.5	37.9	38.4	38.3	36.5	38.1	38.0
% 1 / week	28.7	28.6	29.0	28.8	28.6	29.1	27.7	28.9	28.0	28.7	28.8	30.1	27.5
% 2-4 / week	18.8	18.3	20.0	17.9	19.7	18.7	19.0	18.8	19.0	18.7	19.7	19.8	18.0
% 5-6 / week	1.5	1.4	1.7	1.3	1.6	1.4	1.6	1.4	1.5	1.4	1.8	1.4	1.5
% ≥ 1 / day	0.4	0.3	0.5	0.3	0.5	0.3	0.5	0.4	0.5	0.4	0.5	0.3	0.4

The UK Biobank (UKB) was assessed over a 5 year time horizon from recruitment. β These models were not sex-specific and so both male and female participants were eligible. ■ In the UKB some colorectal cancer diagnosis included more than one anatomical site. If the cancer encompassed both colon and rectum at the date of first diagnosis it was classified as colorectal and was not included in the subsite analysis (e.g. colon or rectal). ^ Mean values with standard deviation (S.D.). Cancer site was defined by the International Statistical Classification of Disease and Related Health Problems, 10th Revision,[17] colorectal encompassed C18-C20, colon C18 and rectal C19 and C20.

Supplementary Table 6: Comparison of eligible and ineligible female participants in The European Prospective Investigation into Cancer and Nutrition

	EPIC Female	Colditz [5]		Freedman [1]		Shin [7]		Steffen [10 β]		Taylor [11 β]		Wells [3]	
		Eligible	Ineligible	Eligible	Ineligible	Eligible	Ineligible	Eligible	Ineligible	Eligible	Ineligible	Eligible	Ineligible
Number of Participants	343,985	149,164	194,821	80,560	263,425	207,887	136,098	189,023	154,962	84,752	259,233	69,154	274,831
(% of cohort)		(43.4)	(56.6)	(23.4)	(76.6)	(60.4)	(39.6)	(55.0)	(45.0)	(24.6)	(75.4)	(20.1)	(79.9)
Colorectal Cases	2,010			714	1,296			533	316	411	1,599	422	1,588
(C.I.R. /100,000 years)	(61.8)			(94.5)	(51.9)			(57.6)	(41.7)	(51.2)	(65.4)	(64.4)	(61.2)
Colon	1,312	744	568					341	205				
(C.I.R. /100,000 years)	(40.4)	(52.6)	(30.9)					(36.9)	(27.1)				
Rectal	698					217	86	192	111				
(C.I.R. /100,000 years)	(21.5)					(21.3)	(12.9)	(20.8)	(14.7)				
Follow-up time (years)	3,250,144	1,414,842	1,835,302	755,233	2,494,912	1,017,063	665,275	924,823	757,515	803,450	2,446,694	655,623	2,594,521
Age^													
% with no data	0.0	0.0	0.0	0.0	0.0	0.0	0.0	0.0	0.0	0.0	0.0	0.0	0.0
Of those with data	50.9	53.7	48.7	57.9	48.8	53.6	46.7	53.5	47.7	52.6	50.3	51.9	50.6
(S.D.)	(9.9)	(7.0)	(11.1)	(4.9)	(10.0)	(7.2)	(11.7)	(7.2)	(11.6)	(7.0)	(10.6)	(7.3)	(10.4)
BMI kg/m^2^													
% with no data	25.6	0.0	45.1	0.0	33.4	0.2	64.3	0.0	56.7	46.0	18.9	0.0	32.0
Of those with data	25.5	25.6	25.2	26.4	25.0	25.8	24.0	25.8	24.4	26.2	25.3	26.3	25.2
(S.D.)	(4.6)	(4.5)	(4.8)	(4.7)	(4.5)	(4.6)	(4.3)	(4.6)	(4.4)	(4.9)	(4.6)	(4.7)	(4.6)
Smoking status													
% with no data	2.6	0.7	4.0	0.8	3.1	1.3	4.5	0.0	5.7	4.6	1.9	0.0	3.2
Of those with data:													
% Never	57.0	57.1	56.9	64.1	54.7	57.4	56.3	57.5	56.3	70.5	52.7	66.1	54.6
% Previous	23.1	23.9	22.4	20.6	23.8	23.0	23.2	22.5	23.8	18.7	24.5	18.8	24.2
% Current	20.0	19.0	20.7	15.3	21.4	19.6	20.5	20.0	20.0	10.8	22.9	15.1	21.2
Alcohol status													
% with no data	24.6	6.9	38.2	2.7	31.3	17.0	36.1	17.2	33.7	4.6	31.1	0.3	30.7
Of those with data:													
% Never	10.5	9.9	11.2	11.5	10.1	10.5	10.4	11.0	9.7	16.3	7.9	13.6	9.4
% Previous	4.9	5.7	3.9	6.3	4.3	5.6	3.5	5.1	4.6	8.0	3.5	6.9	4.2
% Current	84.6	84.4	84.9	82.2	85.7	83.9	86.2	83.9	85.8	75.7	88.7	79.5	86.5
Vigorous recreation activity (MET hours / week) ^													
% with no data	30.1	19.4	38.4	0.0	39.4	26.9	35.1	27.1	33.8	8.3	37.3	1.1	37.4
Of those with data	12.6	11.2	14.1	11.7	13.1	11.7	14.2	11.6	14.0	10.1	13.8	8.8	14.1
(S.D.)	(23.8)	(22.2)	(25.2)	(23.1)	(24.1)	(22.9)	(25.1)	(22.9)	(24.8)	(22.8)	(24.1)	(19.4)	(25.1)
Processed meat consumption (g/day) ^													
% with no data	1.0	0.0	1.7	0.0	1.3	0.0	2.4	0.0	2.1	2.4	0.5	0.0	1.2
Of those with data	27.4	26.0	28.5	24.0	28.5	25.9	29.8	26.1	29.1	29.5	26.7	27.7	27.3
(S.D.)	(25.8)	(24.6)	(26.6)	(24.8)	(26.0)	(25.3)	(26.3)	(25.6)	(25.8)	(24.9)	(26.0)	(28.6)	(25.0)

The European Prospective Investigation into Cancer and Nutrition (EPIC) was assessed over a 10 year time horizon from recruitment, with the exception of Shin et al [7] and Steffen et al [10] which were assessed over 5 years. [β] These models were not sex-specific and so both male and female participants were eligible. ^ Mean values with standard deviation (S.D.). Cancer site was defined by the International Classification of Diseases for Oncology, 2nd revision (ICD-O-2),[18] colorectal encompassed C18-C20, colon C18 and rectal C19 and C20.

Supplementary Table 7: Published and derived coefficients for the validated risk prediction models

Author	Colditz [5]	Driver [8]	Freedman [1]	Ma [9]	Shin [7]	Steffen [10]	Taylor [11]
Sex						-0.254<br>CRC (women vs men)<br>-0.053<br>CC (women vs men)<br>-0.702<br>RC (women vs men)	
Age		0.81<br>Male CRC (50-59)<br>1.48<br>Male CRC (60-69)<br>1.83<br>Male CRC (≥70)	-0.60<br>Female DCC (>65)	0.08<br>Male CRC (year)<br><br>0.085<br>Male CC (year)<br><br>0.067<br>Male RC (year)	0.10<br>Male right CC (age-mean age, years)<br>0<br>Male right CC (age-mean age ^{2}, years^2)<br><br>0.08<br>Female RC (age-mean age, years)<br>0<br>Female RC (age – mean age^2, years^2)	0.065<br>CRC (year)<br>0.073<br>CC (year)<br>0.045<br>RC (year)	
Height	0.26<br>Male CC (≥ 5 feet 10 inches)<br>Female CC (≥ 5 feet 7 inches)				0.06<br>Female RC (>151 - ≤155 cm)<br>0.14<br>Female RC (>155 - ≤158 cm)<br>0.21<br>Female RC (>158 cm)		

Body mass index	0.41<br>CC (≥27 kg/m^2)	0.23<br>Male CRC (25.0-29.9 kg/m^2)<br>0.48<br>Male CRC (≥30 kg/m^2)	0.23<br>Male PCC (25.0-≤30 kg/m^2)<br>0.46<br>Male PCC (>30 kg/m^2)<br><br>0.32<br>Male DCC (25.0-≤30 kg/m^2)<br>0.64<br>Male DCC (>30 kg/m^2)<br>0.08<br>Female DCC (>30 kg/m^2)<br><br>0.34<br>Female RC (>30 kg/m^2)	0.047<br>Male CRC (kg/m^2)<br><br>0.049<br>Male CC (kg/m^2)	0.10<br>Male right CC (≥ 25.0 kg/m^2)	0.020<br>CRC (kg/m^2)<br>0.027<br>CC (kg/m^2)<br>0.006<br>RC (kg/m^2)	
Family history of cancer	0.59<br>CC (sibling or parent with colon cancer)		0.59<br>Male PCC (1 first degree relative with CRC)<br>1.19<br>Male PCC (≥ 2 first degree relatives with CRC)<br><br>0.52<br>Male DCC (1 first degree relative with CRC)<br>1.03<br>Male DCC (≥ 2 first degree relatives with CRC)<br><br>0.40<br>Male RC (1 first degree relative with CRC)		0.25<br>Male right CC (family history of cancer)		-0.12 ■<br>(no affected first-degree relative)<br>0.65 ■<br>(1 affected first-degree relative)<br>1.10 ■<br>(2 affected first-degree relatives)<br>1.49 ■<br>(3 affected first-degree relative)

Family history of cancer (continued)		0.41<br>Female PCC (1 first degree relative with CRC)<br>0.82<br>Female PCC (≥ 2 first degree relatives with CRC)<br>0.37<br><br>Female DCC (1 first degree relative with CRC)<br>0.74<br>Female DCC (≥ 2 first degree relatives with CRC)<br><br>0.43<br>Female RC (1 first degree relative with CRC)	
Diabetes			0.221<br>CRC (prevalent diabetes)<br>0.086<br>CC (prevalent diabetes)<br>0.480<br>RC (prevalent diabetes)
Screening / Endoscopy	-0.69<br>CC (flexible sigmoidoscopy or FOBT within 10 years)	0.35<br>Male PCC (No Endoscopy in last 10 years)<br>0.57<br>Male PCC (Endoscopy in last 10 years and history of polyps)<br>0.46<br>Male PCC (Endoscopy and polyps unknown)	-0.531<br>CRC (ever had CRC screening)<br>-0.345<br>CC (ever had CRC screening)<br>-0.953<br>RC (ever had CRC screening)

Screening / Endoscopy (continued)	1.04<br>Male DCC (No Endoscopy in last 10 years)<br>0.29<br>Male DCC (Endoscopy in last 10 years and history of polyps)<br>0.96<br>Male DCC (Endoscopy and polyps unknown)<br><br>1.35<br>Male RC (No Endoscopy in last 10 years)<br>0.65<br>Male RC (Endoscopy in last 10 years and history of polyps)<br>-0.67<br>Male RC (Endoscopy and polyps unknown)<br><br>0.60<br>Female PCC (No Endoscopy in last 10 years)<br>0.96<br>Female PCC (Endoscopy in last 10 years and history of polyps)<br>-0.49<br>Female PCC (Endoscopy and polyps unknown)

Screening / Endoscopy (continued)		1.24<br>Female DCC (No Endoscopy in last 10 years)<br>1.47<br>Female DCC (Endoscopy in last 10 years and history of polyps)<br>1.15<br>Female DCC (Endoscopy and polyps unknown)<br><br>1.10<br>Female RC (No Endoscopy in last 10 years)<br>1.16<br>Female RC (Endoscopy in last 10 years and history of polyps)<br>-0.99<br>Female RC (Endoscopy and polyps unknown)
Inflammatory bowel disease	0.41<br>CC (≥ 10 years)	
Oestrogen status		-0.39<br>Female PCC (Negative in last 2 years)<br>-0.73<br>Female DCC (Negative in last 2 years)

Oestrogen status (continued)		-0.40<br>Female RC (Negative in last 2 years)	
BMI- Oestrogen interaction		0.99<br>Female DCC	
Hormone replacement therapy	-0.22<br>Female CC (≥ 5 years)		
Oral Contraceptive Pill	-0.36<br>Female CC (≥ 5 years)		
Non-steroidal anti-inflammatory drugs	-0.36<br>CC (Aspirin daily use ≥ 15 years)	-0.43<br>Male PCC (regular use)<br>-0.34<br>Male DCC (regular use)<br>-0.42<br>Male RC (regular use)<br><br>-0.46<br>Female PCC (regular use)<br>-0.36<br>Female DCC (regular use)<br>-0.36<br>Female RC (regular use)	
Physical Activity	-0.51 *<br>CC (≥ 25 MET-hour/week)	-0.19<br>Male RC (>0 - ≤ 2 h/week)<br>-0.37<br>Male RC (>2 - ≤ 4 h/week)<br>-0.56<br>Male RC (>4 h/week)	-0.019<br>Male CRC (MET-h/day)<br><br>-0.020<br>Male CC (MET-h/day)

Physical Activity (continued)		-0.15<br>Female PCC (>0 - ≤ 2 h/week)<br>-0.29<br>Female PCC (>2 - ≤ 4 h/week)<br><br>-0.43<br>Female RC (>4 h/week)<br>-0.37<br>Female RC (>0 - ≤ 2 h/week)<br>-0.24<br>Female RC (>2 - ≤ 4 h/week)<br>-0.46<br>Female RC (>4 h/week)	-0.020<br>Male RC (MET-h/day)		
Smoking	0.35<br>Male CRC<br>(current/previous smoker)	0.26<br>Male PCC (>0 - <11 cigarettes/day)<br>0.53<br>Male PCC (≥11 - ≤20 cigarettes/day)<br>0.80<br>Male PCC (>20 cigarettes/day)<br><br>-0.51<br>Male PCC (>0 - <15 years smoking)<br>-0.13<br>Male PCC (≥15 - <35 years smoking)	0.071<br>Male CRC (former)<br>0.239<br>Male CRC (current)<br><br>0.186<br>Male CC (former)<br>0.347<br>Male CC (current)		0.240<br>CRC (former)<br>0.269<br>CRC (current)<br><br>0.109<br>CC (former)<br>0.165<br>CC (current)<br><br>0.536<br>RC (former)<br>0.476<br>RC (current)

Smoking (continued)			-0.40<br>Male PCC (≥35 years smoking)			
Alcohol	0.34 *<br>CC (> 7 servings/week)	0.31<br>Male CRC (≥once/week)		-0.163<br>Male CRC (never)<br>0.358<br>Male CRC (regular, <300 g/w)<br>0.659<br>Male CRC (regular ≥ 300g/week)<br><br>-0.140<br>Male CC (never)<br>0.419<br>Male CC (regular, <300 g/week)<br>0.655<br>Male CC (regular ≥ 300g/week)<br><br>-0.094<br>Male RC (never)<br>0.365<br>Male RC (regular, <300 g/week)<br>0.745<br>Male RC (regular ≥ 300g/week)	0.13<br>Male right CC (1-14.9 g/day)<br>0.18<br>Male right CC (15-24.9 g/day)<br>0.22<br>Male right CC (≥25 g/day)<br><br>0<br>Female RC (1-14.9 g/day)<br>0.39<br>Female RC (≥ 15 g/day)	0.080<br>CRC (drinks/day)<br><br>0.095<br>CC (drinks/day)<br>0.051<br>RC (drinks/day)

Red Meat/Meat	0.41*<br>CC (≥ 1.2 servings/day)		0.09<br>Male right CC (2-3 times / week)<br>0.21<br>Male right CC (≥ 4 times / week)<br><br>0.03<br>Female RC (2-3 times / week)<br>0.33<br>Female RC (≥ 4 times / week)
Vegetables	-0.36<br>CC (≥ 3 servings/day)	-0.54<br>Male PCC (≥5 servings /day)<br><br>-0.33<br>Female PCC (≥5 servings /day)	
Vitamins	-0.69<br>CC (Regular multivitamin use)		

CRC = colorectal cancer, CC = colon cancer, PCC = proximal colon cancer, DCC = distal colon cancer, RC = rectal cancer, g = grams, h = hours, kg = kilogram, m^2 = meters squared, MET = Metabolic equivalent task. Where odds ratios, hazard ratios or relative risks were published these were transformed into coefficients using the natural log. * Coefficient values were calculated from relative risks published in a subsequent validation paper or the variable definition from the validation was utilised [13]. ■ Coefficient values were calculated from relative risks in a preceding paper published by several of the authors [12]. The reference categories for categorical variables are not included. Please see the original publications for further description of the variables definitions and model construction. Permission was not obtained to present the previously unpublished coefficients of the Wells et al [3] model and so the values are omitted from this table.

References:

1. Freedman AN, Slattery ML, Ballard-Barbash R, et al. Colorectal cancer risk prediction tool for white men and women without known susceptibility. *J Clin Oncol* 2009;27(5):686-93. doi: 10.1200/jco.2008.17.4797 [published Online First: 2008/12/31]
2. National Cancer Institute Division of Cancer Epidemiology and Genetics. Colon Cancer Risk Assessment - SAS Macro [Available from: https://dceg.cancer.gov/tools/risk-assessment/ccratsasmacro accessed 23rd June 2017.
3. Wells BJ, Kattan MW, Cooper GS, et al. Colorectal cancer predicted risk online (CRC-PRO) calculator using data from the multi-ethnic cohort study. *J Am Board Fam Med* 2014;27(1):42-55. doi: 10.3122/jabfm.2014.01.130040 [published Online First: 2014/01/07]
4. Wells BJ, Kattan MW, Cooper GS, et al. Colorectal Cancer - 10 year Predicted Risk Online (CRC-PRO) 2014 [Available from: http://riskcalc.org/ColorectalCancer/ accessed 20th June 2017.
5. Colditz GA, Atwood KA, Emmons K, et al. Harvard report on cancer prevention volume 4: Harvard Cancer Risk Index. Risk Index Working Group, Harvard Center for Cancer Prevention. *Cancer Causes Control* 2000;11(6):477-88. [published Online First: 2000/07/06]
6. Haftenberger M, Friedenreich C, Lahmann PH, et al. Anthropometric Data in the EPIC Study. In: International Agency for Research on Cancer, ed. Unpublished Internal Document, 2015.
7. Shin A, Joo J, Yang HR, et al. Risk prediction model for colorectal cancer: National Health Insurance Corporation study, Korea. *PLoS One* 2014;9(2):e88079. doi: 10.1371/journal.pone.0088079 [published Online First: 2014/02/18]
8. Driver JA, Gaziano JM, Gelber RP, et al. Development of a risk score for colorectal cancer in men. *Am J Med* 2007;120(3):257-63. doi: 10.1016/j.amjmed.2006.05.055 [published Online First: 2007/03/14]
9. Ma E, Sasazuki S, Iwasaki M, et al. 10-Year risk of colorectal cancer: development and validation of a prediction model in middle-aged Japanese men. *Cancer Epidemiol* 2010;34(5):534-41. doi: 10.1016/j.canep.2010.04.021 [published Online First: 2010/06/18]
10. Steffen A, MacInnis RJ, Joshy G, et al. Development and validation of a risk score predicting risk of colorectal cancer. *Cancer Epidemiol Biomarkers Prev* 2014;23(11):2543-52. doi: 10.1158/1055-9965.EPI-14-0206
11. Taylor DP, Stoddard GJ, Burt RW, et al. How well does family history predict who will get colorectal cancer? Implications for cancer screening and counseling. *Genet Med* 2011;13(5):385-91. doi: 10.1097/GIM.0b013e3182064384 [published Online First: 2011/01/29]
12. Taylor DP, Burt RW, Williams MS, et al. Population-based family history-specific risks for colorectal cancer: a constellation approach. *Gastroenterology* 2010;138(3):877-85.
13. Kim DJ, Rockhill B, Colditz GA. Validation of the Harvard Cancer Risk Index: a prediction tool for individual cancer risk. *Journal of Clinical Epidemiology* 2004;57(4):332-40.

14. International Physical Activity Questionnaire. Guidelines for Data Processing and Analysis of the International Physical Activity Questionnaire (IPAQ) - Short and Long Forms, 2005.
15. Uk biobank. Data_Field 20161 [Available from: http://biobank.ctsu.ox.ac.uk/crystal/field.cgi?id=20161 accessed 10th July 2017.
16. Department of Health. Alcohol Units, a brief guide. In: Department of Health, ed., 2008.
17. UK Biobank. Data-Field 40006, Notes [Available from: http://biobank.ctsu.ox.ac.uk/crystal/field.cgi?id=40006 accessed 3rd July 2017.
18. European Prospective Investigation into Cancer and Nutrition. Guidelines_Cancer_Followup_2015_Nov. Unpublished internal document.
