# Supplementary figures and images for: Comparison of prognostic models to predict the occurrence of colorectal cancer in asymptomatic individuals: a systematic literature review and external validation in the EPIC and UK Biobank prospective cohort studies
Source: Gut. 2018 Apr 3;68(4):672–83. doi: 10.1136/gutjnl-2017-315730 (PMC6580880; doi:10.1136/gutjnl-2017-315730)

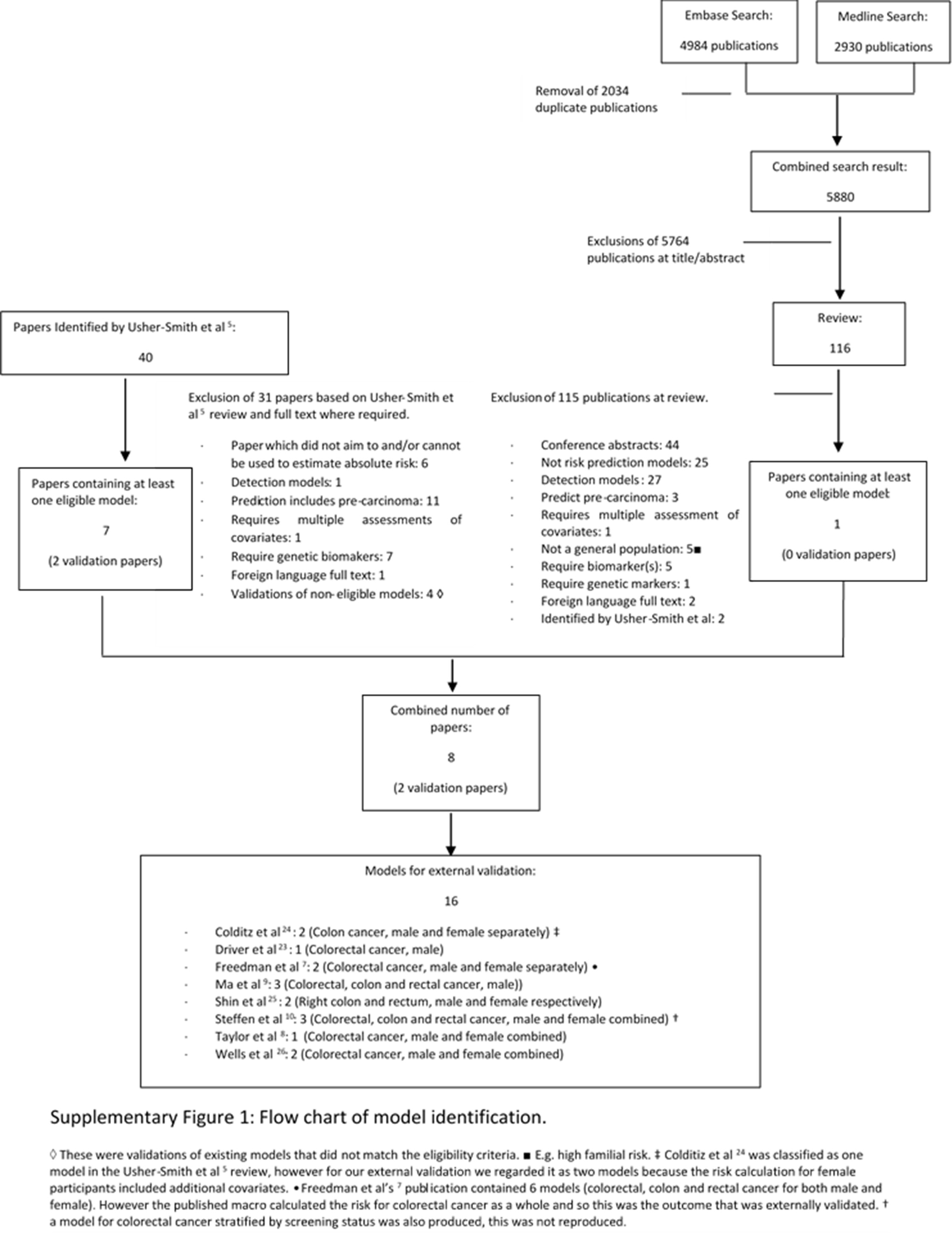

Supplement: Supplementary file 2 [file gutjnl-2017-315730supp002.jpg]

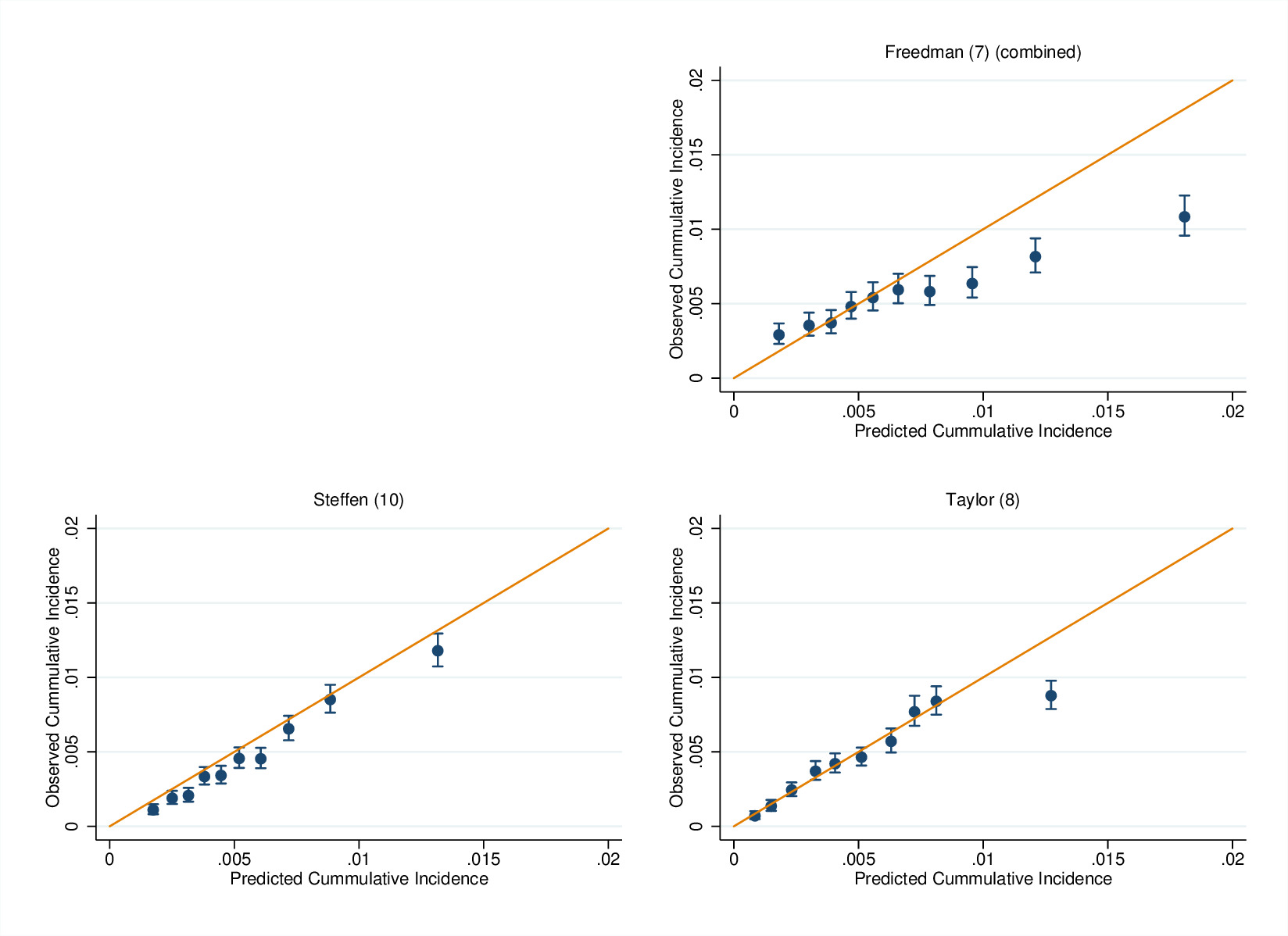

Supplement: Supplementary file 3 [file gutjnl-2017-315730supp003.jpg]

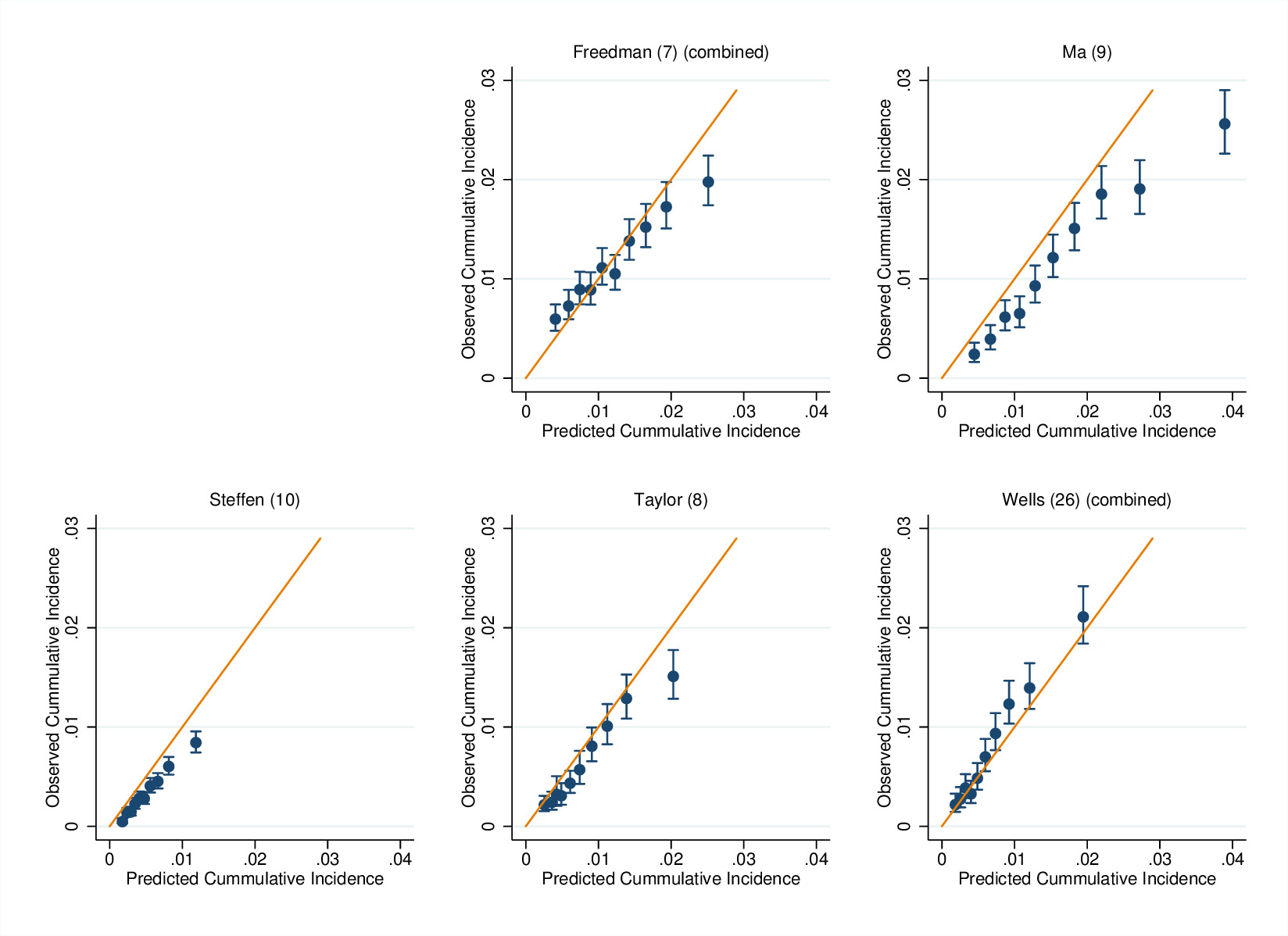

Supplement: Supplementary file 4 [file gutjnl-2017-315730supp004.jpg]

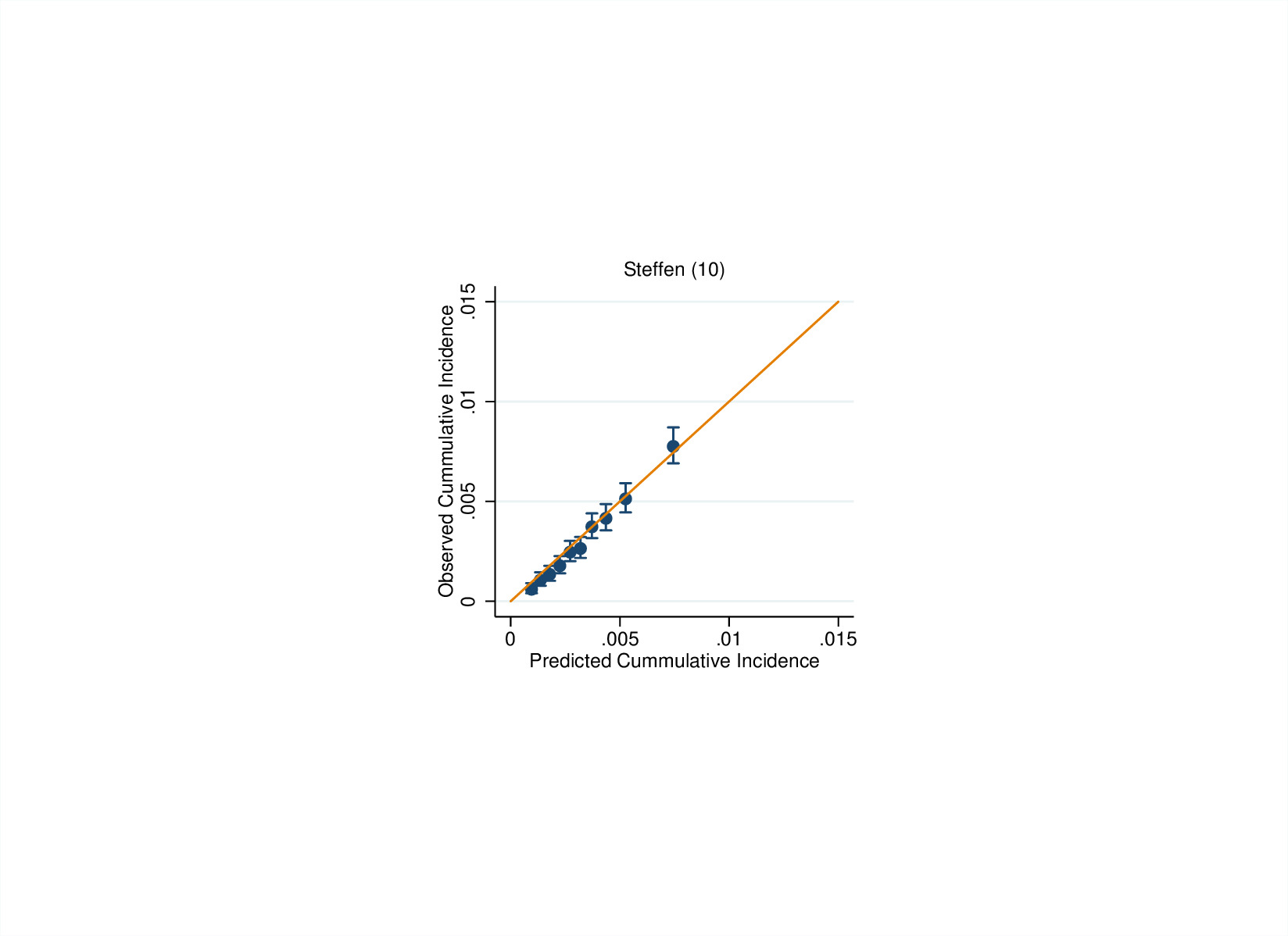

Supplement: Supplementary file 5 [file gutjnl-2017-315730supp005.jpg]

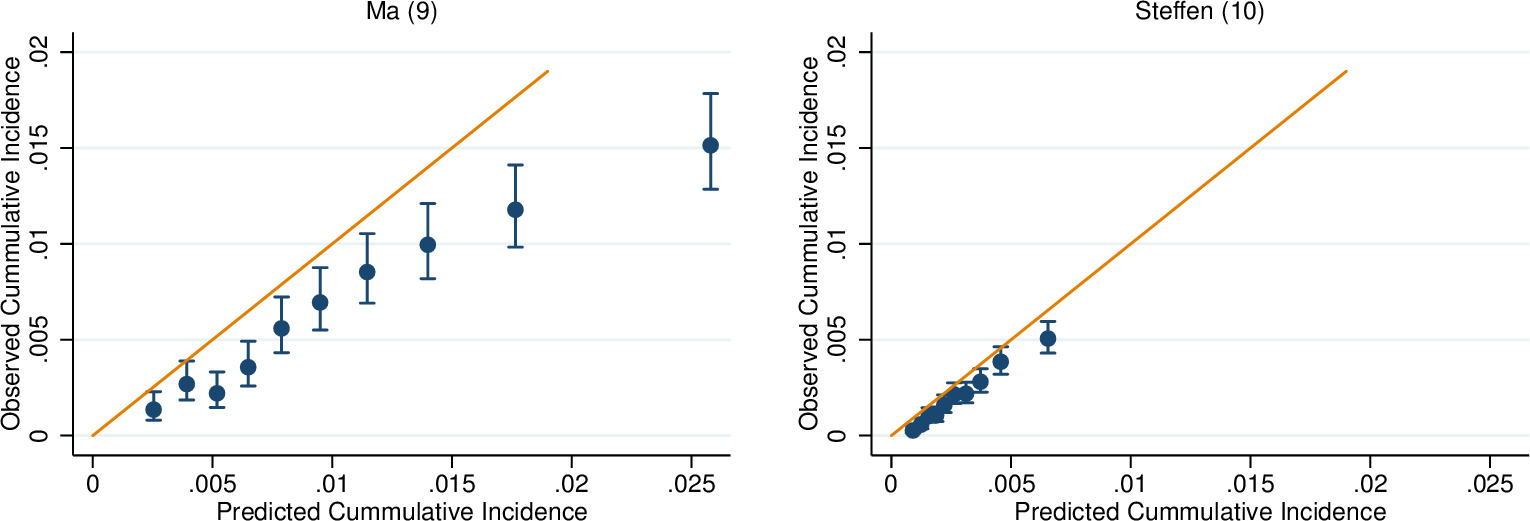

Supplement: Supplementary file 6 [file gutjnl-2017-315730supp006.jpg]

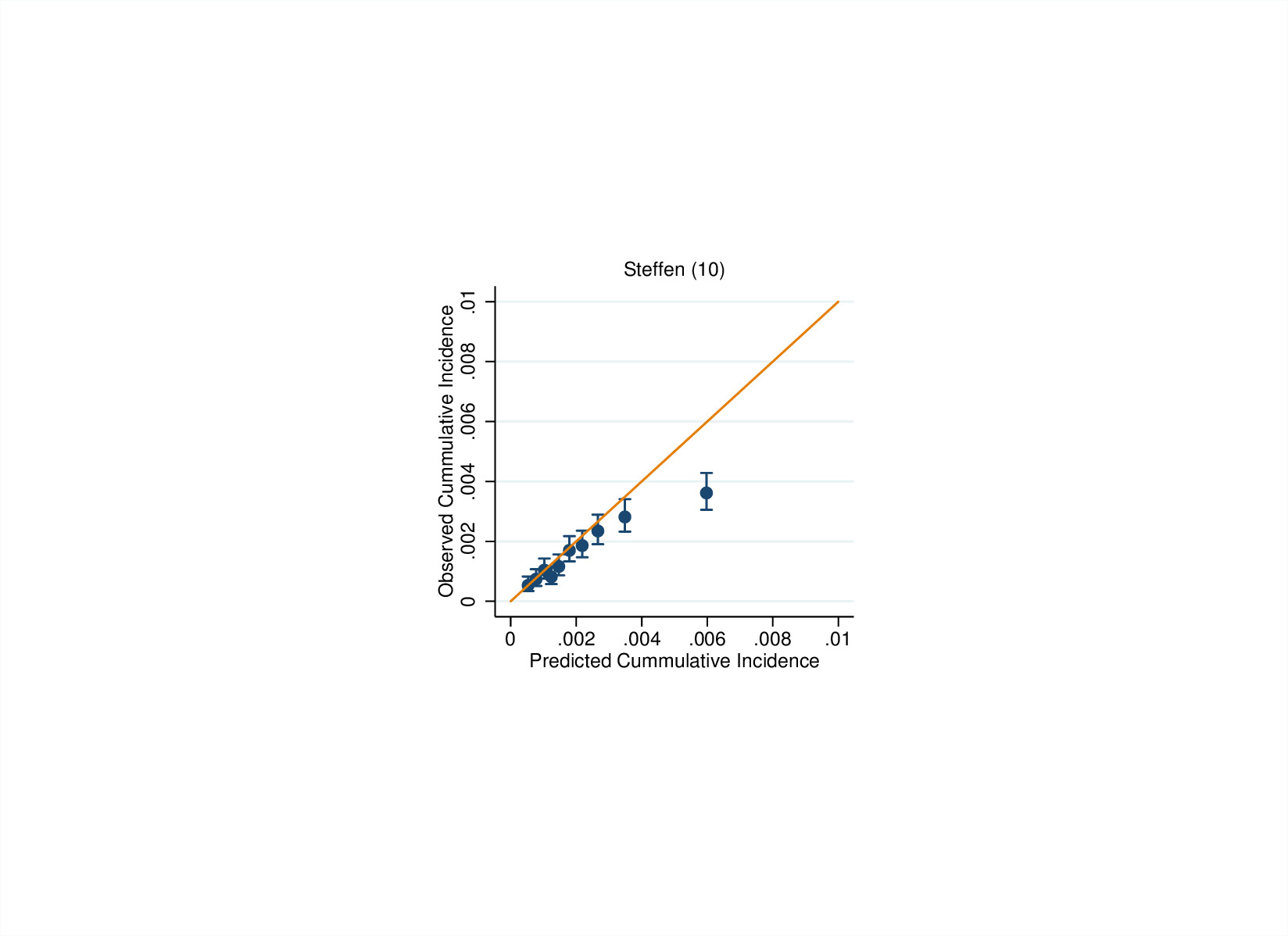

Supplement: Supplementary file 7 [file gutjnl-2017-315730supp007.jpg]

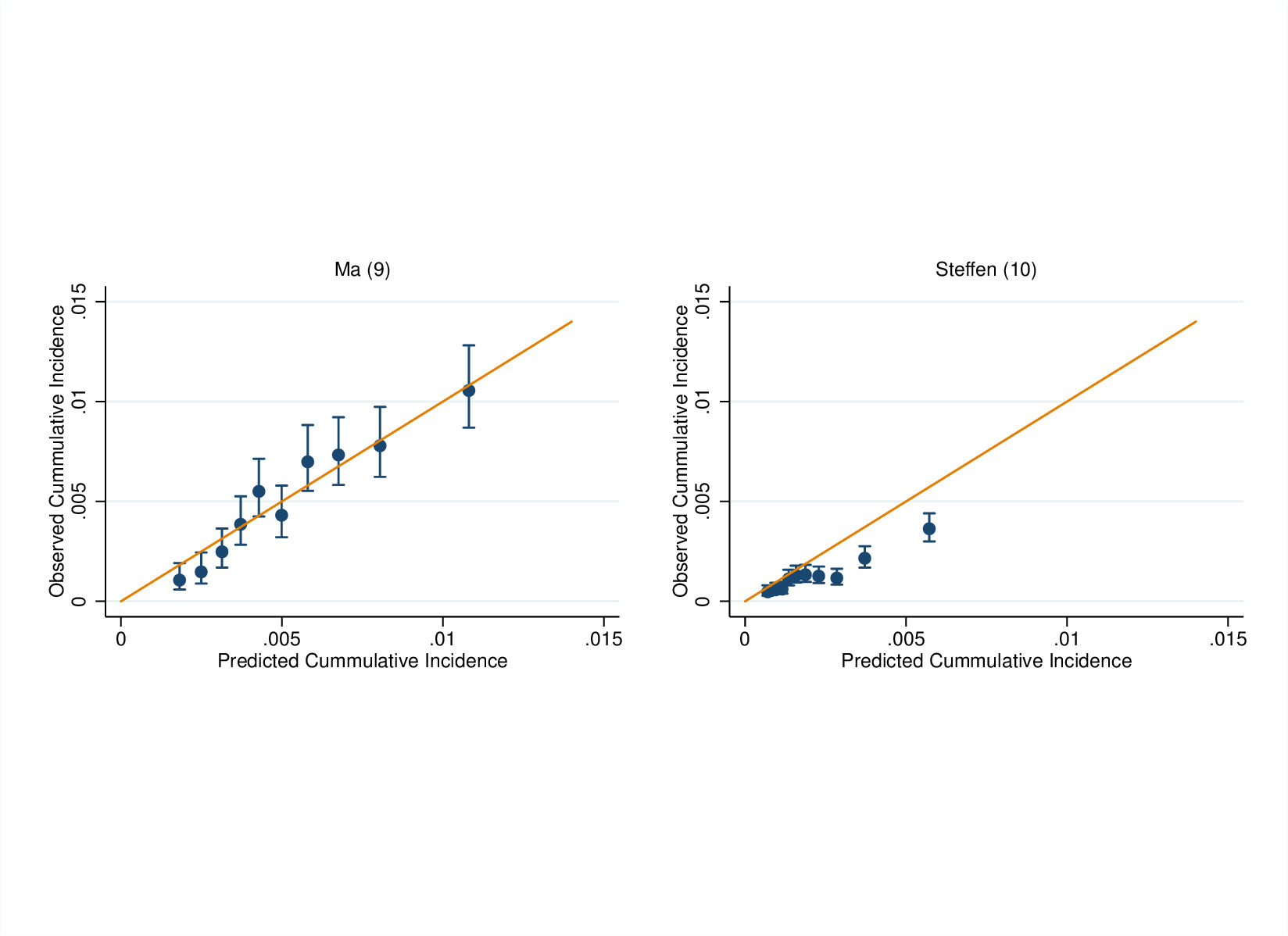

Supplement: Supplementary file 8 [file gutjnl-2017-315730supp008.jpg]
